# Thalidomide as an Effective Treatment in Sideroblastic Anemia, Immunodeficiency, Periodic Fevers, and Developmental Delay (SIFD)

**DOI:** 10.1007/s10875-023-01441-7

**Published:** 2023-02-02

**Authors:** Yan Li, Mengyue Deng, Tongxin Han, Wenxiu Mo, Huawei Mao

**Affiliations:** Department of Immunology, Ministry of Education Key Laboratory of Major Diseases in Children, Beijing Children’s Hospital, National Center for Children’s Health, Capital Medical University, No. 56 Nanlishi Road, Beijing, 100045 China

**Keywords:** SIFD, TRNT1, Thalidomide, Therapy, Novel mutation, China

## Abstract

**Purpose:**

Sideroblastic anemia, immunodeficiency, periodic fevers, and developmental delay (SIFD) is an autosomal recessive syndrome caused by biallelic loss-of-function variant of tRNA nucleotidyl transferase 1 (TRNT1). Efficacious methods to treat SIFD are lacking. We identified two novel mutations in TRNT1 and an efficacious and novel therapy for SIFD.

**Methods:**

We retrospectively summarized the clinical records of two patients with SIFD from different families and reviewed all published cases of SIFD.

**Results:**

Both patients had periodic fever, developmental delay, rash, microcytic anemia, and B cell lymphopenia with infections. Whole-exome sequencing of patient 1 identified a previously unreported homozygous mutation of TRNT1 (c.706G > A/p.Glu236Lys). He received intravenous immunoglobulin (IVIG) replacement and antibiotics, but died at 1 year of age. Gene testing in patient 2 revealed compound heterozygous mutations (c.907C > G/p.Gln303Glu and c.88A > G/p.Met30Val) in TRNT1, the former of which is a novel mutation. Periodic fever was controlled in the first month after adalimumab therapy and IVIG replacement, but recurred in the second month. Adalimumab was discontinued and replaced with thalidomide, which controlled the periodic fever and normalized inflammatory markers effectively. A retrospective analysis of reported cases revealed 69 patients with SIFD carrying 46 mutations. The male: female ratio was 1: 1, and the mean age of onset was 3.0 months. The most common clinical manifestations in patients with SIFD were microcytic anemia (82.6%), hypogammaglobulinemia/B cell lymphopenia (75.4%), periodic fever (66.7%), and developmental delay (60.0%). In addition to the typical tetralogy, SIFD features several heterogeneous symptoms involving multiple systems. Corticosteroids, immunosuppressants, and anakinra have low efficacy, whereas etanercept suppressed fever and improved anemia in reports. Bone-marrow transplantation can be used to treat severe SIFD, but carries a high risk. In total, 28.2% (20/71) of reported patients died, mainly because of multi-organ failure. Biallelic mutations located in exon1-intron5 lead to more severe phenotypes and higher mortality. Furthermore, 15.5% (11/71) patients survived to adulthood. The symptoms could be resolved spontaneously in five patients.

**Conclusions:**

Thalidomide can control the inflammation of SIFD and represents a new treatment for SIFD.

**Supplementary Information:**

The online version contains supplementary material available at 10.1007/s10875-023-01441-7.

## Introduction

Sideroblastic anemia, immunodeficiency, periodic fevers, and developmental delay (SIFD) is an autosomal recessive syndrome caused by biallelic loss-of-function variant of tRNA nucleotidyl transferase 1 (TRNT1) [1]. TRNT1 is an important enzyme for the post-transcriptional modification of transfer RNAs (tRNAs) in the mitochondria and cytoplasm of cells. TRNT1 adds cytosine/cytosine/adenine (CCA) trinucleotides to the 3ʹ-end of newly synthesized tRNAs. The CCA terminus is primarily responsible for tRNA localization on the ribosome, and it can terminate protein translation, which is important for protein formation [2]. In addition, TRNT1 can recognize and repair damaged tRNAs and mark structurally unstable tRNA for degradation. TRNT1 is also involved in mitochondrial metabolism, clearance of abnormal proteins, and oxidative stress [1]. SIFD was first reported in 2013 by Wiseman and colleagues [3]. As of 1 November 2022, 71 cases have been reported globally, including the two cases described in present study.

There are significant variations in clinical manifestations and the prognosis of SIFD, and these differences are related to the mutation site [1]. Approximately one-third of patients with SIFD die, and most surviving patients have sequelae, which suggests that the disease lead to a poor outcome. Treatments for SIFD include blood transfusion, intravenous immunoglobulin (IVIG) replacement, corticosteroids, and anakinra, but their efficacy has been shown to be suboptimal. Conversely, etanercept can relieve fever and anemia in patients with SIFD [4–11]. Bone-marrow transplantation (BMT) can be another efficacious treatment option in selected patients, but transplantation-related mortality is high [3, 5, 8, 12, 13].

Here, we report on two unrelated Chinese patients with SIFD. They had two novel mutation sites, so the SIFD genotype was extended in our study. For the first time, we used thalidomide in the treatment of a patient with SIFD, and the result was good. Our data suggest that thalidomide is a new and effective treatment for SIFD. We also summarized the clinical phenotypes, genetic mutation sites, treatments, and prognosis of all patients reported with SIFD so far, which we hope will improve understanding of this rare disease.

## Materials and Methods

### Patients

Written informed consent was obtained from the parents of both participants. This study was conducted in accordance with the tenets of the Declaration of Helsinki 1964 and its later amendments. The study protocol was approved by the ethics committee of Beijing Children’s Hospital (Beijing, China).

Two children admitted to our department between 1 April 2021 and 1 November 2022 were diagnosed with SIFD. The medical history of the children was complete. The duration of follow-up after initial treatment was > 12 months. The clinical manifestations, laboratory data, treatments, and prognoses of both patients were collected and analyzed. The patients were diagnosed with SIFD according to their medical history, diagnostic test result, and genetic data.

### Genetic Testing

Peripheral blood was collected from members of the patients’ families after obtaining written informed consent. Samples of whole blood were sent to MyGenostics (Beijing, China) and subjected to whole-exome sequencing (WES). The pathogenicity of the mutations was assessed following the American College of Medical Genetics and Genomics guidelines. Mutations in *TRNT1* were confirmed using Sanger sequencing. Briefly, genomic DNA was extracted from peripheral blood mononuclear cells using a QIAamp DNA Mini Kit (Qiagen, Shanghai, China). Polymerase chain reaction (PCR) was performed to amplify the genomic *TRNT1* gene. The PCR products were sent for Sanger sequencing.

### Protein Structure Modeling

All protein models were visualized with the molecular graphics program PyMol. The homology modeling of TRNT1 structure was performed based on the crystal structure of human CCA-adding enzyme (PDB ID: 1OU5). The mutant structure of TRNT1 was generated using the PyMol mutagenesis tool. Hydrophobic surfaces were colored based on a normalized hydrophobicity scale by running the python script “Color_h” (https://pymolwiki.org/index.php/ Color_h). The electrostatic potentials were performed in PyMol using the vacuum electrostatics function.

### Literature Review

A literature review of SIFD was undertaken using databases (PubMed, Web of Science, and EMBASE). The search terms were “Sideroblastic anemia, immunodeficiency, periodic fevers and developmental delay,” “SIFD,” and “TRNT1.” All studies (articles, reviews, letters, or case reports) published online before 1 December 2022 were eligible for review.

## Results

### Clinical Manifestations of Patients

Patient 1 was born to non-consanguineous, healthy parents of Chinese descent following an uneventful pregnancy. He had developmental delay and intermittent eczema after birth. He developed recurrent fever at 1.5 months of age, occurred once a week for 3 days in each instance. Antibiotics were not efficacious. Vomiting and diarrhea occurred simultaneously during episodes of fever. Laboratory tests revealed a normal leukocyte count, increased levels of C-reactive protein (CRP) and ferritin, and an increased erythrocyte sedimentation rate (ESR). The symptoms disappeared and levels of inflammatory markers decreased during afebrile intervals. Microcytic hypochromic anemia was detected with normal reticulocytes at an age of 2.5 months, but sideroblastic anemia was not detected in peripheral blood or bone marrow during the same period. Examination of immune function during fever at 3 months of age is listed in Table 1. Humoral immune function revealed a reduced level of immunoglobulin (Ig) A levels and normal levels of IgG and IgM. The test results of lymphocyte subtypes showed a normal number of T lymphocytes, reduced number of B cells, and an increased number of natural killer (NK) cells. The interleukin (IL)-6 level was increased. The patient experienced severe infections, including pneumonia (adenovirus and extended-spectrum-β-lactamase-producing *Klebsiella pneumoniae* in sputum), enteritis (*Clostridium difficile* in stools), and urinary-tract infection (extended-spectrum-β-lactamase-producing *K. pneumoniae* in urine). High-resolution computed tomography of the lungs revealed bilateral inflammatory changes. Magnetic resonance imaging (MRI) of the brain disclosed widening of bilateral ventricles, cisterna magna, and left extracerebellar space. The patient’s oldest sister had identical clinical manifestations. She died of severe pneumonia at 2 years of age without undergoing genetic testing.

**Table 1:** Laboratory examination of two cases of SIFD

Patient number (age, mo)	P1 (3)	P2 (59)
Routine blood examination		
White blood cell (WBC) count × 10^9^/L	5.8 (8.0–12.0)↓	10.3 (4.0–10.0)
Neutrophil ratio (NEUT) %	27.8 (30.0–40.0)↓	75.9 (35.0–65.0)
Hemoglobin (HGB) g/L	78.0 (110.0–160.0)↓	73.0 (110.0–160.0)↓
Mean corpuscular volume (MCV) fL	66.0 (73.0–100.0)↓	55.4 (80.0–100.0)↓
Mean corpuscular hemoglobin (MCH) pg	19.0 (26.0–32.0)↓	17.1 (27.4–34.0)↓
Mean corpuscular hemoglobin concentration (MCHC) g/L	280.0 (320.0–410.0)↓	308.0 (320.0–360.0)↓
Platelet (PLT) count × 10^9^/L	266.0 (100.0–400.0)	368.0 (100.0–400.0)
Inflammatory index		
C-reactive protein (CRP) mg/L	27.0 (< 8.0)↑	261.0 (< 8.0)↑
Erythrocyte sedimentation rate (ESR) mm/h	17.0 (0.0–15.0)↑	34.0 (0.0–20.0)↑
Ferritin ng/mL	960.3 (28.0–397.0)↑	564.1 (6.0–159.0)↑
Serum immunoglobulin level		
IgA g/L	0.03 (0.05–0.60)↓	< 0.1 (0.4–1.8)↓
IgM g/L	0.4 (0.1-–0.7)	0.1 (0.4–1.8)↓
IgG g/L	2.9 (2.8–7.5)	1.7 (5.0–13.0)↓
Lymphocyte subsets		
CD3 + T cells/uL	3336.1 (766.0–4068.0)	10686.0 (1775.0–3953.0)↑
CD3 + CD4 + T cells/uL	1778.6 (1890.0–2988.0)↓	2469.0 (948.0–2477.0)
CD3 + CD8 + T cells/uL	1505.1 (658.0–1276.0)↑	2057.0 (531.0–1521.0)↑
CD3 + , TCRγδ + cells/uL		4257.0 (128.0–520.0)↑
CD4 + /CD8 +	1.2 (1.1–2.0)	1.2 (1.1–2.5)
CD16 + CD56 + NK cells/uL	1345.6 (211.0–722.0)↑	1621.0 (241.0–978.0)↑
CD19 + B cells/uL	245.9 (667.0–2044.0)↓	125.0 (537.0–1464.0)↓
Cytokines		
IL-6 pg/mL	47.0 (< 7.0)↑	222.2 (≤ 5.4)↑
IL-8 pg/mL		51.2 (≤ 20.6)↑
IFN-α pg/mL		< 2.4 (≤ 8.5)
IFN-γ pg/mL		5236.4 (≤ 23.1)↑
MIP-1α pg/mL		23.4 (≤ 21.0)↑
IL-1RA pg/mL		13,024.0 (≤ 2171.0)↑
MCP-1 pg/mL		335.0 (≤ 127.0)↑
IP-10 pg/mL		116.5 (≤ 172)
Others		
Serum iron umol/L		26.3 (9.0–21.5)

Abbreviations: *WBC*, white blood cell; *NEUT*, neutrophil ratio; *HGB*, hemoglobin; *MCV*, mean corpuscular volume; *MCH*, mean corpuscular hemoglobin; *MCHC*, mean corpuscular hemoglobin concentration; *PLT*, platelet; *CRP*, C-reactive protein; *ESR*, erythrocyte sedimentation rate

**Fig. 1:**
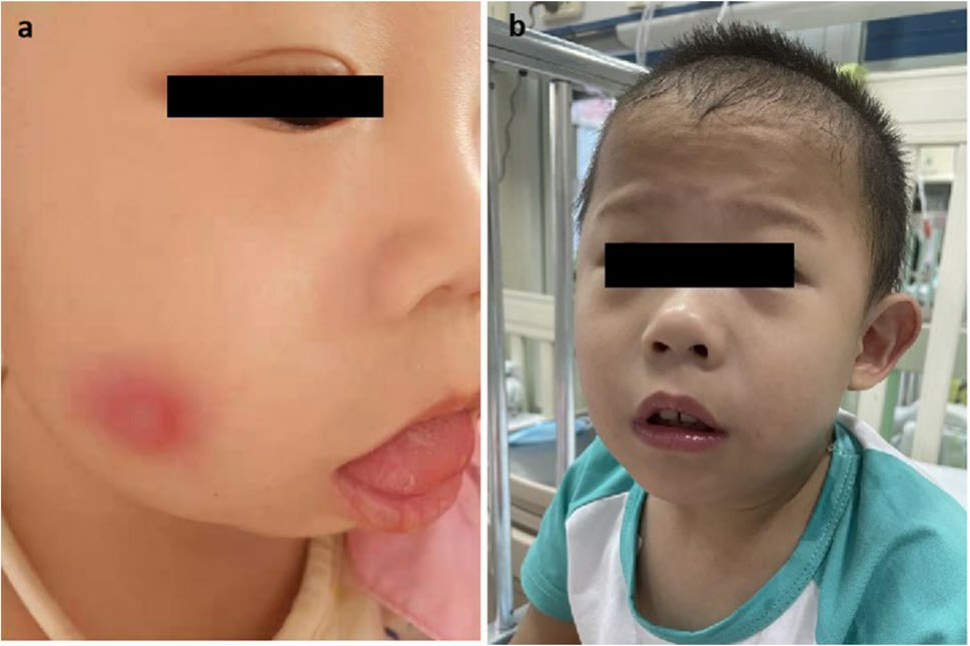
Clinical manifestation of patient 1 we reported. (**a**) Erythematous rash with partial ulceration of patient 2. (**b**) Facial dysmorphism of patient 2 presenting with mild facial dysmorphism and protruding backed nose

Patient 2 was born to unrelated Chinese parents after a normal pregnancy. She developed periodic high fever 17 h after birth accompanied by vomiting and diarrhea that did not respond to antibiotics. Fever persisted for 5 days, followed by 7 days of normal body temperature. Her inflammatory indices (CRP level, ESR, and ferritin level) were increased during fever and decreased in the afebrile interval. Non-infectious erythematous rash with partial ulceration appeared during periods of fever (Fig. 1a). The pathology of the erythema included degeneration and necrosis in the epidermis and subcutaneous tissue. At the age of 1 month, moderate-to-severe microcytic hypochromic anemia and thrombocytopenia were observed, and examination of bone marrow revealed occasional nucleated red cells with possible sideroblastic granules (but no ringed sideroblasts) and normal megakaryocytes. Conduction in the auditory nerve was aberrant. MRI of the brain revealed mild dilatation of bilateral lateral ventricles. The patient coughed intermittently and experienced pneumonia 2–3 times a year, which was cured by antibiotics. Sputum culture disclosed *K. pneumoniae*. Her gross motor and language skills were delayed. She had mild facial dysmorphism (Fig. 1b) and a failure to thrive. She was admitted to our department when she was 5 years of age. Testing of immune function was done during hospitalization, and the results indicated hypogammaglobulinemia, B cell lymphopenia, an increased number of T lymphocytes and NK cells, and a significantly increased number of γδT cells. In addition, levels of IL-6, IL-8, and interferon (IFN)-γ were increased, but levels of IFN-α and IFN-γ-induced protein 10 (IP-10) were normal (Table 1).

### Identification of TRNT1 Mutations in the Patients

**Fig. 2:**
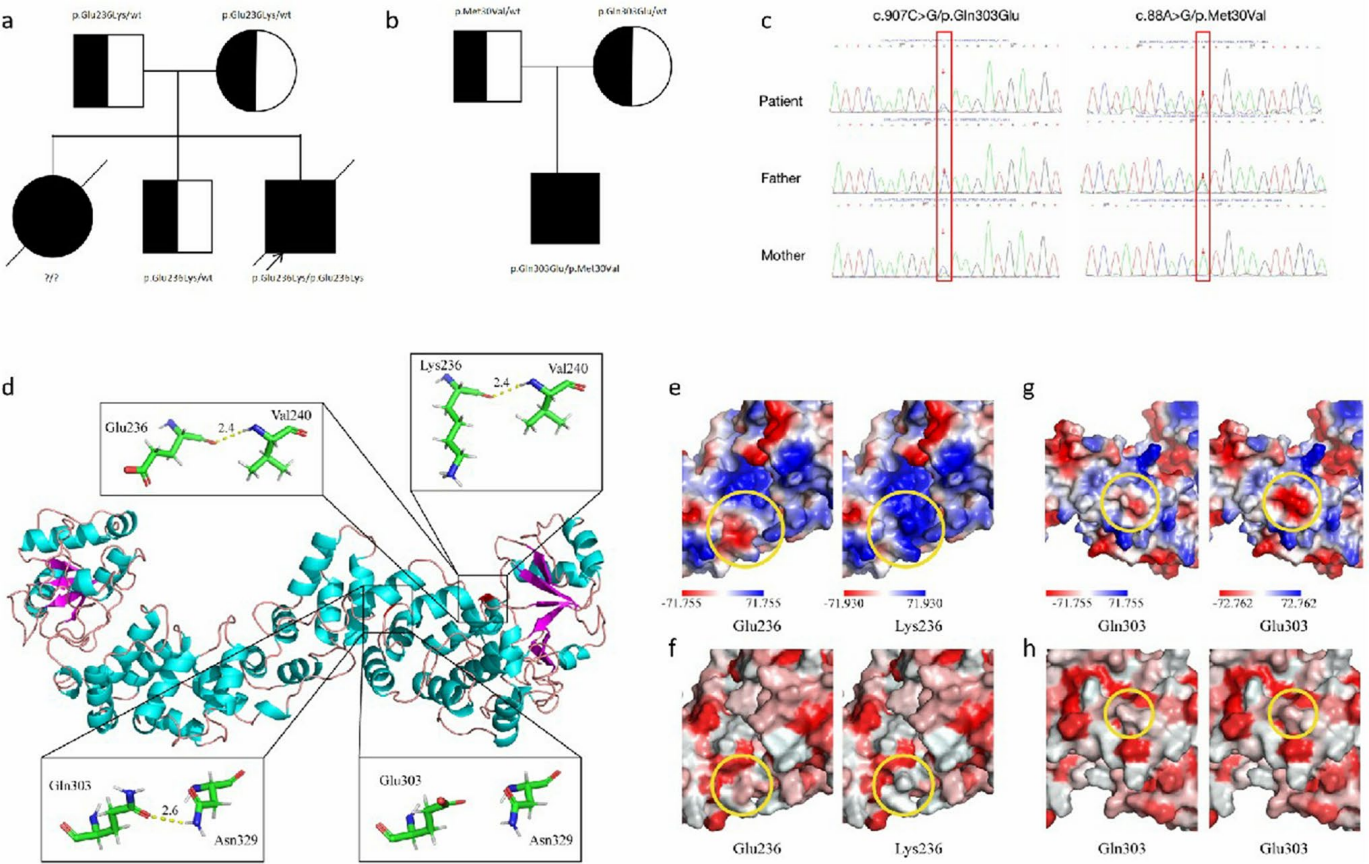
TRNT1 mutations in our two patients. (a and b) Pedigree of family 1 (**a**) and family 2 (**b**). Circles, females; squares, males; filled symbol, affected subject; half-filled symbols, the heterozygous carriers; slash lines, dead individuals. (**c**) Sanger sequencing results of *TRNT1* in patient 2 and his parents. (**d**) Crystal structure of the TRNT1 was shown in cartoon representation and colored by the secondary structure. Magnified pictures displayed the sticks of Glu236 and Val240 residues (top-left), Lys236 and Val240 residues (top-right), Gln303 and Asn329 residues (bottom-left), and Glu303 and Asn329 residues (bottom-right). (**e** and **g**) The calculated electrostatic potentials of the TRNT1 WT and mutant were mapped on the surfaces and colored in a gradient from red (negative) to blue (positive). Yellow circles represent the corresponding amino acid. (**f** and **h**) The predicted hydrophobicity of TRNT1 WT and mutant gradient colors from red to white indicate a hydrophobicity scale from high to low. Yellow circles represent the corresponding amino acid

Based on clinical and laboratory information, WES was performed for the two patients and their families. A homozygous missense mutation in exon 6 (c.706G > A and p.Glu236Lys) of the *TRNT1* gene was identified in patient 1. Both his parents and 3-year-old brother were heterozygous mutation carriers of this mutation (Fig. 2a). In addition, the mutation was not found in the gnomAD (v2.1.1), ClinVar, or Human Gene Mutation Database. Compound heterozygous mutations of *TRNT1* (c.907C > G, p.Gln303Glu and c.88A > G, p.Met30Val) were found in patient 2, and these mutations were inherited from her mother and father, respectively (Fig. 2b and c). Of these, p.Met30Val was known as a pathogenic mutation, whereas p.Gln303Glu has not been reported previously. Further pathogenicity analysis by MutationTaster and GERP + found that the p.Gln303Glu mutation was predicted to be pathogenic.

In silico studies were performed to evaluate the impacts of these mutations on TRNT1 structure. The amino acid at position 236 of TRNT1 was located in the PcnB domain and near a highly conserved position, and the mutation resulted in a substitution of glutamic acid (Glu) with lysine (Lys). The mutation did not change the number of hydrogen bond or the distance between Glu236 and Val240 (Fig. 2d). However, it introduced an opposite charge and changed from negative (Glu) to positive (Lys), which may disrupted interaction with other molecules (Fig. 2e). Besides, the mutation also decreased local hydrophobic interactions, which could alter the physicochemical properties of the enzyme (Fig. 2f). The mutant residue Gln303 was located on the surface of PcnB domain in the TRNT1 protein. The hydrogen bond (yellow dotted line) distance between Gln303 and Asn329 was 2.6 Å, whereas the Glu303 mutation did not form a hydrogen bond with Asn329 (Fig. 2d). Beyond that, according to the electrostatic potential calculation, the mutation, charge changed from neutral (Gln) to negative (Glu), may cause a clash and repulsion with neighboring residues (Fig. 2g). The change in surface hydrophobicity potential was not significant (Fig. 2h). Collectively, these results strongly indicated that these mutations might alter the structure and function of TRNT1.

### Treatment and Outcome of the Patients

Patient 1 was diagnosed with SIFD and treated with IVIG replacement once a month. He died of severe infection at 1 year of age.

**Fig. 3:**
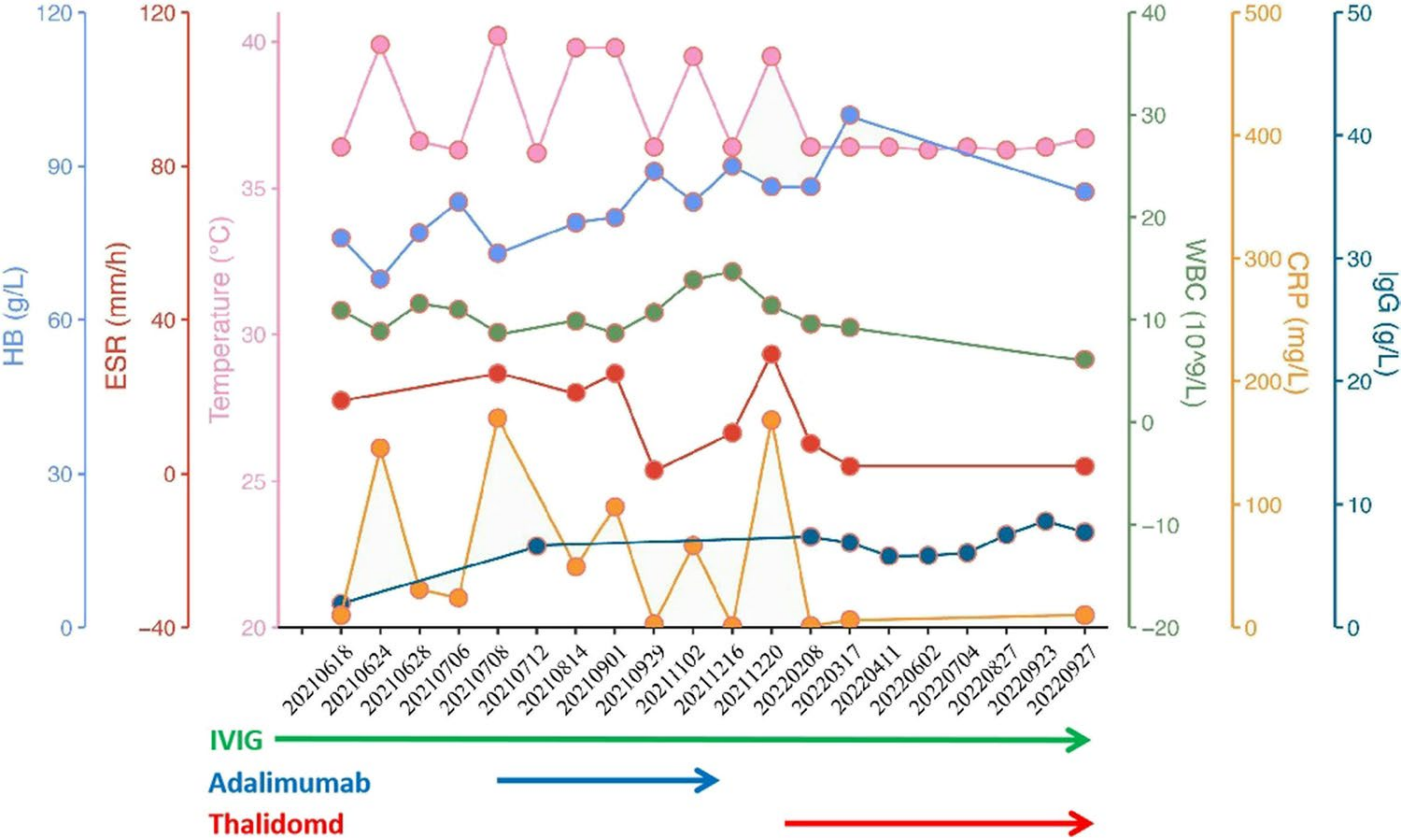
Schematic diagram of clinical features and lab data of patient 2 following treatment

Patient 2 received blood transfusions, IVIG replacement, empiric antibiotics, and systemic corticosteroids, but the efficacy of those treatments was poor. At the age of 5 years, she was treated with adalimumab combined with IVIG replacement (400 mg/kg/m) in our hospital. At the beginning of treatment, her temperature was normal. Adalimumab was discontinued 5 months later because of the reappearance of periodic fever after 1 month of treatment. Thalidomide was administered together with IVIG supplementation therapy. After 11 months of observation, the patient no longer developed periodic fever and infection, and the inflammatory indicators were normal (Fig. 3). Changes in immune function and cytokines releases were monitored before and after thalidomide administration (Table 2). We documented normalization of the number of T lymphocytes, γδT cells, NK cells, and IgG levels, an increased number of B cells, and reduced levels of IL-6 and IFN-γ. Initially, she was receiving IVIG (400 mg/kg, i.v.) supplementation every month. For nearly 6 months, IVIG was given (i.v.) once every 50–60 days, but the trough concentration of IgG before each infusion was maintained at a normal level. The hemoglobin level increased to 100 g/L after 3 months of thalidomide treatment, but decreased again upon recent retesting. The influence of thalidomide on microcytic anemia, growth, and development requires further long-term observation, but its curative effect on SIFD was significant in this patient.

**Table 2:** The changes of immune function and cytokines of patient 2 before and after thalidomide treatment

	Before thalidomide (2021.6.18)	After thalidomide (2022.9.27)
Serum immunoglobulin level		
IgA g/L (0.4–1.8)	< 0.1	0.2
IgM g/L (0.4–1.8)	0.1	0.4
IgG g/L (5–13)	1.7	7.7
Lymphocyte subsets		
CD3 + T cells/uL (1775–3953)	10686.0	2705.0
CD3 + CD4 + T cells/uL (948–2477)	2469.0	1227.0
CD3 + CD8 + T cells/uL (531–1521)	2057.0	1234.0
CD3 + , TCRγδ + cells/uL (128–520)	4257.0	256.0
CD16 + CD56 + NK cells/uL (241–978)	1621.0	493.0
CD19 + B cells/uL (537–1464)	125.0	226.0
Cytokines		
IL-6 pg/mL (≤ 5.4)	222.2	28.1
IFN-α pg/mL (≤ 8.5)	< 2.4	< 2.4
IFN-γ pg/mL (≤ 23.1)	5236.4	117.7

### Review of the Literature on SIFD

To date, 71 cases of SIFD have been reported based on 33 articles (Supplementary Table 1) [3–36]. In those articles, 23 patients had a family history of the disease. Among the 65 patients whose sex was reported, the male: female ratio was 1: 1. Patients developed SIFD as early as the fetal period [13], and the older age of presentation was 40 years [35]. Only one patient had adult-onset SIFD [35]. The mean age of onset was 3.0 (Q1–Q3 = 1.0–7.2) months.

#### Clinical Manifestation

Clinical information was available for 69 of 71 patients. The clinical manifestations and laboratory features of the 69 patients were analyzed (Table 3) [3–36]. The clinical features of SIFD were diverse. Microcytic anemia (82.6%), hypogammaglobulinemia/B cell lymphopenia (75.4%), recurrent fever (66.7%), and developmental delay (60.9%) were the most common manifestations of SIFD. Also, 36.2% (25/69) of patients had concurrent tetralogy. Three patients had none of the typical four manifestations of SIFD stated above [19], and they all had ocular symptoms accompanied by microcytosis and anisocytosis and were finally diagnosed as SIFD by gene sequencing. Microcytic anemia was present in 57 patients, of which 30 had sideroblastic anemia. Microcytosis was observed in four patients [19, 32]. Thrombocytopenia was documented in three patients, and one patient also had leukopenia [12, 36]. Three-quarters of patients with SIFD had hypogammaglobulinemia, B cell lymphopenia, and reduced production of protective antibodies [35]. They were prone to recurrent infections by multiple pathogens in different systems. Infections were present in 43.5% (30/69) of patients. In 2020, Zhou et al. reported a patient with severe coronavirus disease-2019 (COVID-19) who had been healthy in the past. Due to recurrence of severe infection by acute respiratory syndrome-coronavirus-2, genetic testing was conducted and a mutation in *TRNT1* was found [35]. Specific autoantibodies of SIFD patients were all negative. Most of children had fever every 1–4 weeks, with each episode lasting 3–7 days. Developmental delay (another main feature of SIFD) can manifest as intellectual disability as well as disorders in motor development and language development.

**Table 3:** Clinical manifestations and laboratory features of TRNT1 deficiency

Clinical feature	Total	Percent%
Recurrent fever	46/69	66.7
Mucocutaneous/skin	28/69	40.6
Skin edema	9/69	13.0
Erythematous nodule	10/69	14.5
Eczema	3/69	4.3
Oral ulcers	9/69	13.0
Gastrointestinal involvement	28/69	40.6
Vomiting and diarrhea with recurrent fever	21/69	30.4
Crohn disease	1/69	1.4
Protein losing enteropathy	1/69	1.4
Necrotizing enterocolitis	1/69	1.4
Feeding intolerance/TPN dependence	6/69	8.7
Developmental delay	42/69	60.9
Neurological manifestations	27/69	39.1
Seizure	11/69	15.9
Hypotonia	7/69	10.1
Ataxia/cerebellar signs	6/69	8.7
Opsoclonus	5/69	7.2
Musculoskeletal involvement	17/69	24.6
Arthritis	11/69	15.9
Myopathy/myositis	6/69	8.7
Ophthalmologic lesions	28/69	40.6
Retinitis pigmentosa	20/69	29.0
Bilateral cataract	12/69	17.4
Sensorineural deafness	21/69	30.4
Cardiomyopathy	6/69	8.7
Metabolic and endocrine abnormalities	20/69	29.0
Aminoaciduria ± hyperalaninemia	8/69	11.6
Metabolic acidosis	8/69	11.6
Hypoglycemia	2/69	2.9
Fanconi syndrome	2/69	2.9
Thyroiditis	2/69	2.9
Primary hypogonadism	2/69	2.9
GH deficiency	4/69	5.8
Failure to thrive	24/69	34.8
Dysmorphic features	22/69	31.9
Sparse and brittle hair	19/69	27.5
Facial dysmorphisms	12/69	17.4
Microcephaly	5/69	7.2
Infections	30/69	43.5
Upper respiratory tract infections	8/69	11.6
Lower respiratory tract infections	9/69	13.0
Pneumonia	12/69	17.4
Gastroenteritis	8/69	11.6
Urinary tract infections	6/69	8.7
Otitis media	3/69	4.3
Splenomegaly	17/69	24.6
Nephrocalcinosis	3/69	4.3
Elevated liver enzymes	4/69	5.8
Pancreatic insufficiency	5/69	7.2
Hypogammaglobulinemia/B lymphopenia	52/69	75.4
Microcytic anemia/sideroblastic anemia	57/69	82.6
Thrombocytopenia	3/69	4.3

Abbreviations: *TPN*, total parenteral nutrition; *GH*, growth hormone

Vomiting and diarrhea during fever were the most common forms of gastrointestinal involvement in 30.4% (21/69) of patients. In other cases, patients had Crohn’s disease, necrotizing enterocolitis, or protein-losing bowel disease that manifested as food intolerance, gastrointestinal bleeding, chronic constipation, or diarrhea. Mucocutaneous lesions were observed in 40.6% (28/69) of patients, and erythema nodosum was the most common manifestation. Skin biopsies were carried out in four patients with erythema nodosum, which revealed panniculitis [7, 8, 23, 28]. Cutaneous vasculitis [16] and lichen sclerosus etatrophicus [18] were rare skin changes of SIFD, each occurring in one patient, respectively. Cardiomyopathy (one of the causes of death) was detected in 8.7% (6/69) of patients, four of whom were neonatal [3, 6, 13]. Involvement of the nervous system was recorded in 39.1% (27/69) of patients. Seizure, hypotonia, and ataxia/cerebellar signs were the most common symptoms. Leigh encephalopathy was diagnosed in one case [36]. Three patients died of status epilepticus [12, 13, 29]. Ocular involvement was documented in 40.6% (28/69) of patients, and it manifested mainly as reduced night vision and blurred vision. Also, 29% (20/69) of patients were diagnosed with retinitis pigmentosa and 17.4% (12/69) with bilateral cataract, the ocular lesions of which are severe and can progress to blindness [8]. In addition, 30.4% (21/69) of patients had sensorineural deafness, which could necessitate the use of hearing aids [4, 8, 14, 20].

The musculoskeletal system was involved in 24.6% (17/69) of patients. Muscle biopsy was undertaken in four patients with muscle involvement; myositis was indicated in one case [8] and metabolic myopathy in the other three patients [3, 5, 24]. Patients with SIFD may have metabolic abnormalities, including aminoaciduria (± hyperalaninemia), metabolic acidosis, and Fanconi syndrome, as well as endocrine abnormalities, including thyroiditis, growth-hormone deficiency, and hypogonadism. Levels of growth hormone should be evaluated in patients with poor growth. Also, 34.8% (24/69) of patients failed to thrive, which may have been related to chronic inflammation, recurrent infections, long-term anemia, gastrointestinal involvement, or deficiency of growth hormone. Two patients had primary hypogonadism (20,33), and WES identified the same homozygous mutation in *TRNT1* (c.295C > T, p. Arg99Trp). In addition, one patient (c.295C > T, p.Arg99Trp and c.1234C > T, p.Arg412X) had a low levels of sex hormones (22). In addition, 32.4% (22/68) of patients had dysmorphic features, including sparse and brittle hair, facial dysmorphisms (deep-seated eyes, short philtrum, thin upper lip, and a protruding, backed nose), and microcephaly. Seventeen patients had splenomegaly, three cases had nephrocalcinosis, four individuals had increased level of liver enzymes, and five patients had pancreatic insufficiency.

#### Laboratory Parameters

A reduced number of T cells and NK cells has been reported for patients with SIFD [17, 25]. However, our patient 1 in our study showed a normal number of T cells and increased number of NK cells. Patient 2 in the present study had an increased number of T cells and NK cells. An increased number of γδT cells was found in two patients, one of whom was our patient 2. Patients with *TRNT1* mutations tend to have increased levels of cytokines (IL-6, IL-18, IL-12p40, IFN-α, and IFN-γ) and IFN-induced chemokines (IP-10 and monokine induced by IFN-γ) in serum, and expression of IFN-stimulated genes is upregulated [8, 25, 31, 37]. Cytokine levels vary slightly in different patients. Tumor necrosis factor (TNF)-α and IL-1β have been detected in the tissues of SIFD patients with active inflammation [8].

#### Genetics

**Fig. 4:**
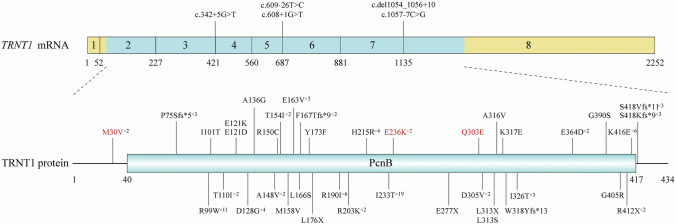
Schematic diagram of TRNT1 mutations reported before

The diagnosis of rare pediatric diseases and familial pediatric diseases caused by genetic defects necessitates use of WES [38]. SIFD is an autosomal recessive disease involving homozygous biallelic mutation (20/68) or compound heterozygous mutation (45/68). A single heterozygous mutations was detected in three patients [5, 9, 34]. In three patients with SIFD, the gene-mutation site was not mentioned in the literature [30, 35]. To date, 48 gene-mutation sites have been discovered worldwide (Fig. 4). Among the reported mutations, c.668 T > C is the most prevalent (14.6%), followed by c.295C > T (10.0%) and c.1057-7C > G (6.2%). Missense mutations are the most common type of mutation, and other mutations include frameshift, splice, and nonsense. We have reported two cases of SIFD: one featured a homozygous TRNT1 mutation and the other featured a compound heterozygous mutation. The two novel mutations (c.706G > A/p.Glu236Lys and c.907C > G/p.Gln303Glu) in our patients were missense mutations.

#### Treatment

The treatment of SIFD is shown in Table 4. Corticosteroids and anakinra have elicited a poor response against SIFD. Etanercept has produced stronger efficacy (10/11, 90.9%), because the drug can control fever and anemia effectively, increase the height and weight of patients, and relieve SIFD-related seizures and arthritis [4–11]. One patient who was being treated with etanercept alone had periodic fever, but combined treatment with colchicine resulted in good control of symptoms [11]. Five patients underwent BMT [3, 5, 8, 12, 13]. Three patients had homozygous mutations (c.569G > T, p.Arg190Ile), two of whom died and the other had improved fever, growth, and development after BMT although hearing loss and retinopathy were present. Three patients had compound heterozygous mutations (c.668 T > C and c.1057-7C > G), two of whom died and the other recovered after BMT. Those data suggest that BMT is an effective treatment for patients with SIFD. However, transplantation-related complications were serious, and three of the five patients died after BMT.

**Table 4:** Treatment reported in SIFD patients

Treatment	Total	Good response	Poor/no response	Not determined
Systemic steroids	15 [6–10, 17, 25, 26, 32]		15 [6–10, 17, 25, 26, 32]	
Anti-IL-1: anakinra	6 [6, 8, 11, 34]		6 [6, 8, 11, 34]	
Anti-TNF-α				
Etanercept	11 [4–11]	10 [4–10]	1 [11]	
Infliximab	1 [8]	1 [8]		
Adalimumab	1^*^		1^*^	
Colchicine	4 [8, 10, 34]		4 [8, 10, 34]	
Methotrexate	1 [19]			1 [19]
Cyclosporine A	1 [8]			1 [8]
Azathioprine	1 [25]	1 [25]		
Thalidomide	1^*^	1^*^		
BMT	5 [3, 5, 8, 12, 13]	2 [3, 5, 12]	3 [5, 8, 13]	

^*^Patient 2 in this article. Abbreviations: *BMT*, bone marrow transplantation

Thalidomide as treatment for SIFD has not been reported. Inflammation was relieved after thalidomide therapy in patient 2 in our study. We speculate that thalidomide may be a new efficacious therapy for SIFD. One patient had severe dermatitis, Crohn’s disease, bilateral cataracts, and B cell lymphopenia. Azathioprine controlled his gastrointestinal symptoms [25]. Supportive treatment is also necessary to improve symptoms, including IVIG replacement, blood transfusions, empiric antibiotics, growth hormone, cochlear implant. cataract surgery, and intraocular lens implantation.

#### Prognosis

**Fig. 5:**
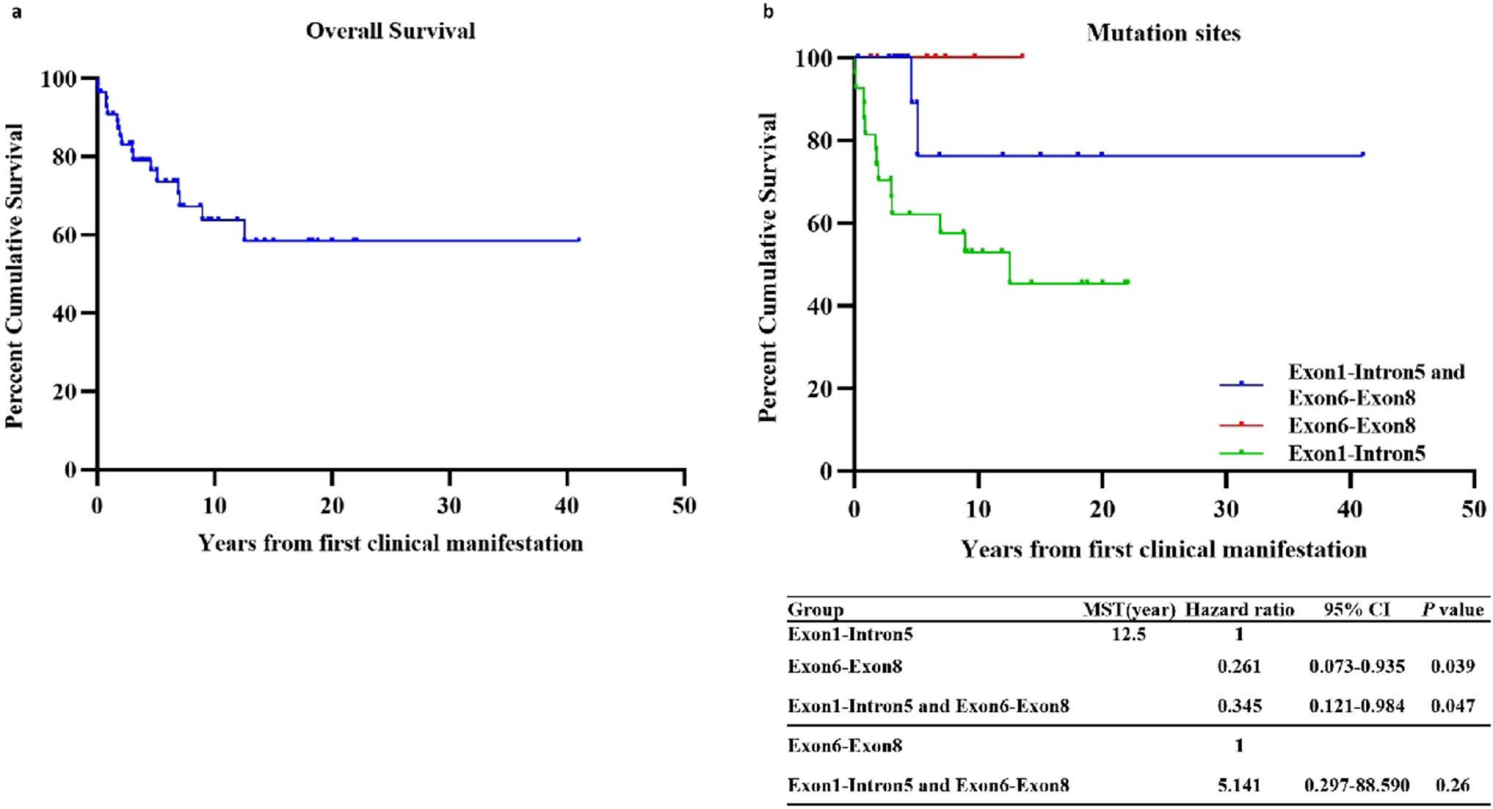
Survival curves of SIFD patients. (**a**) Overall survival of SIFD patients. (**b**) Patients with both mutations located in exon1-intron5 had the highest mortality, and the differences with other two groups were significant (*p* < *0.05*). Abbreviations: *MST*, median survival time

In total, 28.2% (20/71) of patients with SIFD died. The age at death ranged from 2 days to 14 years, and the median age at death was 26.0 (Q1–Q3 = 12.0–56.0) months. The causes of death were multi-organ failure during fever (7/20), post-BMT complications (3/20), cardiac failure (3/20), progressive encephalopathy (3/20), and shock (2/20). Some patients had two or more causes of death (Table 5). Twenty patients developed the first symptoms of SIFD in the neonatal period, of whom 11 died, which suggested that the earlier the onset of SIFD, the higher is the risk of death. Overall cumulative survival of the SIFD patients was 71.2% (Fig. 5a), and the median survival age was 5.7 (Q1–Q3 = 3.0–13.5) years. Mutation sites were associated with prognosis of SIFD. All patients were divided into three groups according to the mutation sites: both mutation sites located in exon1-intron5, one mutation site located in exon1-intron5 and the other located in exon6-exon8, both mutation sites located in exon6-exon8. Patients with both mutations located in exon1-intron5 had the highest mortality, and the differences with other two groups were significant (*p* < *0.05*) (Fig. 5b).

**Table 5:** Cause of death in patients with SIFD

Patient	Allele1/Allele2	Age of onset (m)	Age at death(m)	Reason of death
1	c.668 T > C, p.Ile223Thr c.488A > T, p.Asp163Val	Soon after birth	36	92 days post-BMT
2	c.443C > T, p.Ala148Val c.443C > T, p.Ala148Val	0.6	21	Cardiac arrest during an episode of fever and acute acidosis
3	c.668 T > C, p.Ile223Thr c.218_219ins22, p.V73fs	7	16	Multi-organ failure (pneumonitis; cardiac failure)
4	c.668 T > C, p.Ile223Thr No mutation/deletion detected	Neonatal	25	Cardiac failure secondary to cardiomyopathy
5	c.569G > T, p.Arg190Ile c.569G > T, p.Arg190Ile	7	28	Cardiac failure secondary to cardiomyopathy
6	c.668 T > C, p.Ile223Thr c.1057-7C > G, ?	0.75	56	Shock with severe hypoglycemia and multi-organ failure
7	c.608 + 1G > T, ? c.461C > T, p.Thr154Ile	?	10	Pulmonary hemorrhage post-BMT
8	c.569G > T, p.Arg190Ile c.569G > T, p.Arg190Ile	18	168	Sepsis with multi-organ failure and toxic epidermal necrolysis (attributed to a cephalosporin)
9	c.668 T > C, p.Ile223Thr c.1057-7C > G, ?	Neonatal	61	Multi-organ failure
10	c.668 T > C, p.Ile223Thr c.608 + 1G > T, ?	Soon after birth	40 h	Progressive jaundice, multi-organ failure and intracranial bleeding
11	c.644A > G, p.His215Arg c.644A > G, p.His215Arg	1	84	Multi-organ failure during a febrile episode
12	c.218_219ins22, p.V73fs c.218_219ins22, p.V73fs	7.2	NA	NA
13	c.668 T > C, p.Ile223Thr c.829G > T, p.Glu277	2.4	NA	NA
14	c.329C > T, p.Thr110Ile c.383A > G, p.Asp128Gly	1	108	Staphylococcus aureus sepsis shock
15	c.668 T > C, p.Ile223Thr c.342 + 5G > T, ?	0.75	10	Intractable status epilepticus
16	c.608G > A, p.Arg203Lys c.565 T > C, p.Ile155Thr	3	26	Sepsis-like episode complicated by multi-organ failure
17	c.668 T > C, p.Ile223Thr c.342 + 5G > T, ?	0.5	39	Progressive encephalopathy
18	c.668 T > C, p.Ile223Thr c.608 + 1G > T, ?	Fetus	6	Significant neurological complications soon after BMT, including intractable seizures, and idiopathic pulmonary syndrome
19	Homozygous mutation	36	NA	Status epilepticus and severe hypoxemia leading to brain death
20	c.706G > A, p.Glu236Lys c.706G > A, p.Glu236Lys	1.5	12	Severe infection

Abbreviations: *BMT*, bone marrow transplantation; *NA*, not available

Prompt and efficacious treatment was another factor affecting the prognosis of SIFD. BMT can be used to treat high-risk patients to relieve symptoms and prolong life. In 2018, Giannelou and colleagues were the first to report that TNF inhibitors could be used to treat SIFD, and no deaths were reported since then. Those results indicate that effective treatment can improve the prognosis of patients with SIFD. In addition, two patients were sibling with the same compound heterozygous mutation. One died of septic shock due to *Staphylococcus aureus* infection at the age of 9 years, and the other survived to adulthood, which suggests the importance of preventing and treating infection through IVIG supplementation and antimicrobial therapy. Eleven patients with SIFD survived to adulthood [3, 5, 6, 8, 18, 19, 25, 33, 35, 36] with the eldest patient being 49 years of age. Furthermore, some features of SIFD could resolve spontaneously at the age of 4–9 years, including height, development, anemia [23, 31], immunoglobulin levels [32], fever, canker sores, and rashes [17, 33]. The prognosis of SIFD requires further investigation.

## Discussion

We diagnosed two Chinese patients with SIFD based on their clinical symptoms and genetic data, and two novel mutation sites of *TRNT1* were discovered. We found that thalidomide could be used to treat SIFD. In addition, we summarized the clinical phenotype and genotype of SIFD based on the literature.

*TRNT1* has eight exons and seven introns, which are located on chromosome 3. *TRNT1* mutations damage mitochondrial and cytoplasmic tRNAs in fibroblasts [8, 12], thereby affecting tRNA maturation and protein formation. Microscopy of the bone marrow and skin biopsies has shown massive cellular degeneration with mitochondrial damage and autophagosomes [8]. Mutations in TRNT1 also cause a decrease in respiratory-chain enzymes (RCE) in muscle, leading to impairment of oxidative phosphorylation (OXPHOS) and causing damage to and dysfunction of mitochondria [12, 39]. Giannelou et al. found that damaged mitochondria in fibroblasts lead to an increase in reactive oxygen species (ROS), which could activate the inflammasome-mediated pathway to stimulate inflammation [8]. Patients with TRNT1 mutations also have dysfunction of the ubiquitin–proteasome system (UPS) and the autophagy-lysosome system, which results in dysregulated clearance of damaged mitochondria and abnormal proteins. Accumulation of abnormal proteins can lead to endoplasmic reticulum (ER) stress, which causes increased production of TNF-α [8, 22]. Therefore, the formation of abnormal protein, dysregulated clearance of damaged mitochondria and abnormal proteins, mitochondrial damage, increased production of ROS, ER stress, and activation of inflammatory pathways are possible mechanisms leading to SIFD.

SIFD has the features of autoinflammation, immunodeficiency, and metabolic abnormalities. The clinical manifestations caused by TRNT1 mutations are highly heterogeneous and involve multiple systems. The diagnosis and evaluation of SIFD require comprehensive examination of the heart, nervous system, eyes, ears, bone marrow, immune system, and levels of sex hormones. Eight patients developed symptoms soon after birth and, in one of them, abnormalities were found upon ultrasound during pregnancy, suggesting that *TRNT1* mutations could affect development during the fetal period. However, one patient was diagnosed with recurrent, severe COVID-19 at the age of 40 years and a mutation was found in *TRNT1*, even though he was in good health previously. Hence, SIFD is highly heterogeneous in terms of clinical manifestations, severity, and age of onset.

The inflammation observed in patient 2 in our study improved significantly after thalidomide treatment. The number of B cells was higher than before treatment, and infection did not recur. Application of IVIG was irregular in the later stage of treatment, but the IgG level was maintained to a normal level. We speculate that B cell lymphopenia may be related to inflammation, which affects the maturation of B cells and production of IgG. Frans and coworkers reported a patient suffering from SIFD with an increased number of cluster of differentiation (CD)56^bright^ cells though the total number of NK cells was reduced [25]. CD56^bright^ cells are associated with high levels of IFN- g and TNF-a [40]. However, inconsistent with previous reports, the NK cells in these two patients were elevated. The type of NK cell in our patients may have been CD56^bright^ cells according to their cytokine levels. Our patients had increase level of IL-6 and IFN-γ in serum, a finding that is consistent with the work of Giannelou and colleagues [8]. Activation of the type-I IFN pathway in patients with SIFD has been reported [25, 31]. Some symptoms of SIFD may be attributed to interferonopathy. We can search for new therapeutic targets from this pathway for refractory cases. TNF-α can be detected in tissues with active inflammation. Symptoms are controlled and infiltration of TNF-α is reduced after treatment with TNF inhibitors, thereby suggesting TNF-α to be the main cause of SIFD.

Corticosteroid therapy has achieved only a partial response in patients with SIFD [6–10, 17, 25, 26, 32], which is similar to our findings in patient 2. Corticosteroids may not be first-line therapy for SIFD due to their weak efficacy and serious side-effects. An increased level of IL-1β has been found in tissues[8], but the IL-1 inhibitor anakinra has weak efficacy against SIFD [6, 8, 11, 34]. Those results suggest that IL-1 might not be a major cause of SIFD. TNF inhibitors can be was an effective treatment for most SIFD patients. Only one patient has been reported to show a partial response to treatment using a TNF inhibitor alone [11]. Patient 2 in our study was administered a TNF inhibitor, adalimumab, which is an antibody against TNF-α, good response was observed in the first month before the disease recurred. Therefore, treatment using TNF inhibitors may be limited to only a few patient. For the first time, we used thalidomide to treat SIFD. Patient 2 had a normal temperature, normal inflammatory indices, reduced level of cytokines, and an increased number of B cells after thalidomide therapy. She was stable without recurrence of SIFD, suggesting that thalidomide is efficacious against SIFD. Thalidomide can inhibit TNF-α release from peripheral mononuclear blood cells and macrophages, reduce the production of IL-6 and IFN-γ, and interfere with induction of the nuclear factor-kappa B (NF-κB) pathway by TNF-α or IL-1β [41]. These may be the reasons why thalidomide can counteract SIFD efficaciously. BMT can relieve symptoms in high-risk patients with SIFD, but the mortality rate is relatively high [3, 5, 8, 12, 13], which may be related to the young age at the time of BMT and importance of the organs involved.

The severity and prognosis of SIFD vary and are related to the time of onset, organ involvement, site and type of mutation, and treatment. About half of patients with a neonatal onset died, which may be related to the susceptibility of cardiac involvement, multi-organ failure, and fetal edema in the newborn. The long-term survival of SIFD is related to the mutation sites. Slade et al. speculated that the “mild” phenotype was associated with mutations at N- or C-terminal sites, whereas the “severe” phenotype was related to mutations in central catalytic sites [1]. We found that patients with biallelic mutations located in exon1-intron5 had more severe symptoms and carried a higher mortality risk of death. Efficacious treatments also improved the prognosis of SIFD and reduced the risk of death. In addition, we found that some symptoms of SIFD could resolve spontaneously. Surviving patients had sequelae such as developmental delay, sensorineural deafness, and reduced vision.

## Conclusions

We identified thalidomide as a new treatment that can control inflammation in patients suffering from SIFD. Larger study cohorts and longer observation periods are needed to clarify the efficacy of thalidomide. In the future, we will explore the biological basis of SIFD, clarify the relationship between genotypes and phenotypes, and investigate the mechanism action of thalidomide. An early diagnosis, comprehensive evaluation, and efficacious treatment can improve the prognosis of SIFD.

## Supplementary Information

Below is the link to the electronic supplementary material.

Supplementary file1 (DOCX 86 KB)

## Data Availability

The datasets analyzed during the current study are available from the corresponding author on reasonable request.
