# Supplementary material for: Thalidomide as an Effective Treatment in Sideroblastic Anemia, Immunodeficiency, Periodic Fevers, and Developmental Delay (SIFD)
Source: J Clin Immunol. 2023 Feb 2;43(4):780–93. doi: 10.1007/s10875-023-01441-7 (PMC9893968; doi:10.1007/s10875-023-01441-7)
Supplement: Supplementary file 1 — Supplementary file1 (DOCX 86 KB) [file 10875_2023_1441_MOESM1_ESM.docx]

**Table 1. Clinical characteristics, genotype, treatment and outcome of 71 SIFD patients reported to date**

| References | Patient | Allele1 Allele2 | Ethnicity | Consanguinity | Family history | Gender | Age of onset(m) | Recurrent fever | Dysmorphic features | Failure to thrive | Developmental delay | Rash and mucosa | Sensorineural deafness | Gastrointestinal involvement* | Splenomegaly | Eyes |
| --- | --- | --- | --- | --- | --- | --- | --- | --- | --- | --- | --- | --- | --- | --- | --- | --- |
| 6 | 1 | c.608G>A,p.Arg203Lys c.1246A>G,p.Lys416Glu | Italian | No | No | F | 0.75 | 十 | 十(facial dysmorphisms, brittle hair, microcephaly, sunken eyes, protruding backed nose) | 十 |  |  | 十 | 十 | 十 | 十(posterior subcapsular cataract) |
| 6 | 2 | c.938delT,p.Leu313fs c.1246A>G,p.Lys416Glu | Italian | No | No | F | 4 | 十 | 十(facial dysmorphisms, brittle hair, microcephaly) | 十 | 十 | 十(painless subcutaneous nodules) | 十 |  | 十 | 十(bilateral cataract) |
| 14 | 3 | c.1057-7C>G,? c.1092A>T,p.Glu364Asp | Asian/European | No | Yes(P3-P4),older | F | 8 | 十 | 十(sparse and brittle hair, trichorrhexis invaginata) | 十 | 十 | 十(severe eczema with recurrent *methicillin-resistant Staphylococcus aureus (MRSA)* skin infections) |  | 十 |  |  |
| 14 | 4 | c.1057-7C>G,? c.1092A>T,p.Glu364Asp | Asian/European | No | Yes(P3-P4),younger | M | 12 |  | 十(frontal bossing and flat helices, sparse and brittle hair) | 十 | 十 |  | 十 |  |  |  |
| 15 | 5 | c.498_501delATTT,p.Phe167fs c.947C>T,p.Ala316Vall | NA | No | No | F | 2 | 十 | 十(brittle and sparse hair, long nose with prominent columella, hypoplastic nasal wings, short philtrum, high implant of ears) |  | 十 | 十(thin skin with venous reticulum on the trunk) |  |  |  | 十(bilateral cataracts, blue sclerae, strabismus, enophthalmus, hypotelorism) |
| 16 | 6 | c.88A>G,p.Met30Val c.363G>T,p.Glu121Asp | China | No | No | M | 6 | 一 |  | 一 | 一 |  |  |  |  |  |
| 16 | 7 | c.302T>C,p.Ile101Thr c.1234C>T,p.Arg412* | China | No | No | M | 135 |  |  | 十 |  | 十(repeated erythema multiforme on both lower limbs, accompanied by tenderness, chilblain-like lesions, numbness, stiffness, Raynaud’s phenomenon that occurred repeatedly in both hands,oral ulcers, pathological analysis of the skin revealed vasculitis) |  |  |  |  |
| 17 | 8 | c.525delT,p.Leu176X c.938T>C,p.Leu313Ser | China | No | No | F | 7 | 十 |  |  | 一 | 十(subcutaneous mass in the right shoulder and left waist) |  |  |  |  |
| 3,5,12 | 9 | c.569G>T,p.Arg190Ile c.569G>T,p.Arg190Ile | South Asian (Pakistani) | Yes | Yes(P9-P10),older | F | 18 | 十 |  |  | 十 | lchthyotic skin changes | 十 |  |  | 十(retinitis pigmentosa) |
| 3,5,12 | 10 | c.569G>T,p.Arg190Ile c.569G>T,p.Arg190Ile | South Asian (Pakistani) | Yes | Yes(P9-P10),younger | M | 2 | 十 |  |  | 十 |  | 十 | 十 |  | 十(retinitis pigmentosa) |
| 3,5 | 11 | c.668T>C,p.I223T c.1057-7C>G,? | Caucasian | No | Maybe(had a similarly affected sibling who died at 4 years old without WES) | F | Neonatal | 十 | 十(brittle hair) |  | 十 |  |  |  | 十 |  |
| 3,5 | 12 | c.668T>C,p.Ile223Thr c.1057-7C>G,? | Caucasian | No | No | M | 0.75 | 十 |  |  | 十 |  |  |  | 十 |  |
| 3,5 | 13 | c.668T>C, p.Ile223Thr no mutation/deletion detected | Caucasian | No | No | F | Neonatal | 十 |  |  | 十 |  | 十 |  |  | 十(retinitis pigmentosa) |
| 3,5 | 14 | c.218_219ins22,NA c.668T>C,p.Ile223Thr | Caucasian (spanish) | No | No | M | 7 | 十 |  |  | 十 |  |  |  | 十 |  |
| 3,5 | 15 | c.668T>C,p.Ile223Thr c.668T>C,p.Ile223Thr | Caucasian (Hispanic) | Yes | No | F | 3 | 十 |  |  | 十 |  | 十 |  | 十 |  |
| 3,5 | 16 | c.497T>C,p.Leu166Ser c.461C>T,p.Thr154Ile | Caucasian | No | No | F | 7 | 十 |  |  | 十 |  | 十 |  |  |  |
| 3,5 | 17 | c.569G>T,p.Arg190Ile c.569G>T,p.Arg190Ile | South Asian (Pakistani) | Yes | No | F | 7 | 十 |  |  | 十 |  |  |  |  |  |
| 3,5 | 18 | c.668T>C,p.I223T c.1057-7C>G,? | Caucasian (UK) | No | No | M | 1.75 | 十 |  |  | 十 |  |  |  |  | 十(retinitis pigmentosa) |
| 3,5 | 19 | c.977T>C,p.Ile326Thr c.472A>G,p.Met158Val | Caucasian (Irish/Polish/Lithuanian) | No | No | M | Neonatal | 十 | 十(brittle hair) |  |  |  |  |  |  |  |
| 5 | 20 | c.60811G>T,NA c.461C>T,p.Thr154Ile | Caucasian | No | NA | NA | NA |  |  |  |  |  |  |  |  |  |
| 5 | 21 | c.1246A>G,p.Lys416Glu c.del1054_1056+10,NA | Caucasian | No | Yes(P21-P22) | NA | NA | 十 |  |  | 十 |  |  |  |  |  |
| 5 | 22 | c.1246A>G,p.Lys416Glu c.del1054_1056+10,NA | Caucasian | No | Yes(P21-P22) | NA | NA | 十 |  |  | 十 |  |  |  |  |  |
| 5 | 23 | c.668T>C,p.Ile223Thr c.1142insATGT,p.Trp381fs | Afro-Caribbean | No | NA | NA | NA |  |  |  |  |  |  |  |  |  |
| 5 | 24 | c.668T>C,p.Ile223Thr/ c.1252_1253insA,S418fs | Caucasian | No | NA | NA | NA |  |  |  |  |  |  |  |  |  |
| 8 | 25 | c.644A>G,p.His215Arg c.644A>G,p.His215Arg | Saudi Arabian | Yes | Yes(P25-P26) | F | 1 | 十 |  |  |  | 十(oral ulcers) |  | 十 | 十 |  |
| 8 | 26 | c.644A>G,p.His215Arg c.644A>G,p.His215Arg | Saudi Arabian | Yes | Yes(P25-P26) | F | 24 | 十 | 十(Mild facial syndromic features) | 十 | 十 | 十(oral ulcers) |  | 十 | 十 |  |
| 8 | 27 | c.668T>C,p.Ile223Thr c.488A>T,p.Asp163Val | Mixed European | No | No | M | Soon after birth | 十 |  | 十 | 十 |  |  | pseudoobstruction with ileostomy at birth, necrotizing enterocolitis 6-week-old, feeding intolerance/TPN dependence | 十 | 十(optic nerve atrophy) |
| 8 | 28 | c.295C>T,p.Arg99Trp c.488A>T,p.Asp163Val | Mixed European | No | No | F | 0.75 | 十 | 十(sparse hair, deep seated eyes, long thin tapering fingers) | 十 | 十 | 十(oral mucosa and tongue ulcerations, erythematous nodules during febrile episodes) | 十 | 十，feeding intolerance, chronic constipation, GI biopsy showed acute focal colitis | 十 | 十(early retinal degeneration) |
| 8 | 29 | c.295C>T,p.Arg99Trp c.488A>T,p.Asp163Val | Mixed European | No | No | F | 1.5 | 十 | 十(plagiocephaly, sparse hair, deep seated eyes, long thin tapering fingers) |  | 十 | 十(oral ulcers and tongue swelling, tender inflammatory subcutaneous nodules with overlying skin erythema (scalp, external ear canal, limbs, chest, back) and purpura) | 十 | 十，feeding intolerance, prolonged diarrhea, GI biopsy showed acute and chronic inflammation in the mucosa of the stomach, terminal ileum, colon | 十 | 十(retinitis pigmentosa, retinal degeneration, optic nerve atrophy, hyperopia, bilateral cataracts, myopic astigmatism-legally blind) |
| 8 | 30 | c.329C>T,p.Thr110Ile c.383A>G,p.Asp128Gly | Mixed European | No | Yes(P30-P31) | M | 72 | 十 |  |  | 十 |  |  | 十 | 十 | 十(retinitis pigmentosa, decreased night time vision, hyperopic astigmatism) |
| 8 | 31 | c.329C>T,p.Thr110Ile c.383A>G,p.Asp128Gly | Mixed European | No | Yes(P30-P31) | F | 1 | 十 |  |  | 十 | 十(asymmetric erythema and swelling of digits of the hands and feet, diffuse swelling of the dorsum of the feet, erythema nodosum on lower extremities, skin biopsy showed septal panniculitis, superficial and deep lymphocytic dermatitis |  | 十 |  | 十(retinitis pigmentosa, decreased night time vision) |
| 8 | 32 | c.1246A>G,p.Lys416Glu c.1245_1246insA,p.Ser418Lysfs*9 | Mixed European | No | No | M | 2 | 十 |  | 十 | 十 |  | 十 | 十 |  | 十(bilateral cataracts) |
| 8 | 33 | c.668T>C,p.Ile223Thr c.1245_1246insA,p.Ser418Lysfs*9 | Mixed European | No | No | F | 0.75 | 十 |  |  | 十 |  | 十 | Protein losing enteropathy, feeding intolerance/TPN dependence |  | 十(retinitis pigmentosa) |
| 13 | 34 | c.608+1G>T,? c.668T>C,p.Ile223Thr | NA | No | No | F | Soon after birth |  |  |  |  | 十(a generalised purpuric skin rash) |  |  | 十 |  |
| 13 | 35 | c.608+1G>T,? c.668T>C,p.Ile223Thr | NA | NA | NA | M | Fetus | 一 |  |  | 一 |  |  | meconium ileus, gastric perforation |  |  |
| 11 | 36 | c.383A>G,Asp128Gly c.1168G>A,Gly390Ser | Caucasian | No | No | M | 4 | 十 |  | 十 | 十 | 十(eczema and mouth ulcers) |  | 十 | 十 |  |
| 4 | 37 | c.495_498del,p.F167Tfs*9 c.1246A>G,p.K416E | NA | No | No | F | 6 | 十 |  | 十 | 十 | 十(numerous firm asymptomatic erythematous papules and nodules in trunk and limbs, skin histopathology revealed an intense dermal neutrophilic infiltrate extending to the subcutaneous, with numerous atypical myeloid cells with a high Ki-67 expression) | 十 |  |  | 十(bilateral cataract) |
| 7 | 38 | c.361G>A,p.Glu121Lys c.407C>G,p.Ala136Gly | Brazilian | No | No | F | 1 | 十 |  |  | 一 | 十(oral ulceration，a diffuse painful skin eruption resembling erythema nodosum, skin histopathology revealed septal panniculitis) |  |  |  |  |
| 18 | 39 | C.668T>C,? C.1057-7C>G,? | Caucasian | No | No | F | Infancy | 十 |  |  |  | 十(extensive indurated plaques(abdomen, flanks, back, and thighs) with hyperand hypopigmentation and epidermal atrophy,which eventually become erythematous episodically,the abdominal skin biopsy was consistent with lichen sclerosus etatrophicus (LSA), while the vulvar one showed morphea） | 十 | 十 |  | 十(retinitis pigmentosa) |
| 19 | 40 | c.1246A,p.S418fs c.126_128delAGA,p.E43inframe | NA | NA | No | M | 12 |  |  |  |  |  |  |  |  | 十(retinitis pigmentosa, nyctalopia) |
| 19 | 41 | c.1246A,p.S418fs c.609-26T>C,IVSS | NA | NA | Yes(P41-P42), older | M | child | 一 |  |  |  |  |  |  |  | 十(poor night vision, mild pallor of the optic nerve heads, significant macular edema OU, mildly attenuated of retinal arterioles, diffuse atrophy of the retinal pigment epithelium in the periphery OU, mild generalized depression OU, significant intraretinal cystoid changes and diffuse outer retinal atrophy with extrafoveal preservation OU) |
| 19 | 42 | c.1246A,p.S418fs c.609-26T>C,IVSS | NA | NA | Yes(P41-P42), younger | M | 156 | 一 |  |  |  |  |  |  |  | 十(nyctalopia, locally revealed absent scotopic responses and severely diminished photopic responses OU of electroretinogram performed, mild pallor of the optic nerve heads, significant macular edema OU, mildly attenuated of retinal arterioles, diffuse atrophy OU of retinal pigment epithelium, mild generalized depression OU of visual fields) |
| 20 | 43 | c.295C>T,p.Arg99Trp c.295C>T,p.Arg99Trp | Indian | Yes | Yes(P43-P44-P45) | F | 9 |  | 十(microcephaly, sparse hair) | 十 | 一 |  |  |  |  | 十(posterior subcapsular cataracts reduced night vision, inner retinal dysfunction, bilateral reduced vision, slight optic disc pallor) |
| 20 | 44 | c.295C>T,p.Arg99Trp c.295C>T,p.Arg99Trp | Indian | Yes | Yes(P43-P44-P45) | M | 7 |  | 十(microcephaly, sparse hair) | 十 | 一 |  |  |  |  | 十(posterior subcapsular cataracts, inner retinal dysfunction, mild disc pallor) |
| 20 | 45 | c.295C>T,p.Arg99Trp c.295C>T,p.Arg99Trp | Indian | Yes | Yes(P43-P44-P45) | F | 3 |  | 十(microcephaly, sparse hair) | 十 | 一 |  | 十 |  |  | 十(dense right cataract，mild left posterior subcapsular cataract, inner retinal dysfunction, mild disc pallor) |
| 21 | 46 | c.914A>T,p.Asp305Val c.914A>T,p.Asp305Val | Turkish | Yes | No | M | 6 | 一 | 十(brittle hair) |  | 一 |  |  |  | 十 |  |
| 22 | 47 | c.295C>T,p.Arg99Trp c.1234C>T,p.Arg412X | Japanese | No | No | M | 1 | 十 |  | 十 | 十 |  |  |  |  | 十(cataracts, hypopigmentation) |
| 23 | 48 | c.1213G>A,p.Gly405Arg c.1057-7C>G,? | White | No | NA | F | 2 | 十 |  | 十 |  | 十（erythematous nodule of the limb, skin biopsy showed lobular and septal neutrophilic panniculitis) |  |  |  |  |
| 24 | 49 | c.443C＞T,p.A148V c.443C＞T,p.A148V | Turkish | Yes | No | F | 0.6 |  |  | 十 | 十 |  | 十 |  |  |  |
| 24 | 50 | c.383A>G,p.Asp128Gly c.518A>T,p.Tyr173Phe | Greek-Cypriot origin | No | No | M | NA |  |  |  | 十 |  |  |  |  |  |
| 25 | 51 | c.C295T,p.Arg99Trp c.C295T,p.Arg99Trp | NA | Yes | No | M | 11 |  | 十(mild ptosis, short philtrum, thin upper lip, a low hairline) |  | 十 | 十(severe dermatitis) | 十 | Crohn disease, invasive enterocolitis caused by Campylobacter jejuni |  | 十(bilateral congenital cataracts) |
| 26 | 52 | c.565T>C,p.Ile155Thr c.608G>A,p.Arg203Lys | Italian | No | Maybe(had a similarly affected older brother who died at 9 months old without WES) | M | 3 | 十 |  | 十 | 十 |  |  | 十 |  |  |
| 12 | 53 | c.668T>C,p.Ile223Thr c.342+5G>T,？ | White European | No | Yes(P53-P54),older | F | 0.5 | 十 |  |  | 十 |  |  | 十, chronic gastritis, partial villous atrophy | 十 | 十(retinal dystrophy) |
| 12 | 54 | c.668T>C,p.Ile223Thr c.342+5G>T,? | White European | No | Yes(P53-P54),younger | M | 0.75 | 十 |  |  |  |  |  | 十, poor feeding, loose stools | 十 | 十(retinal pigmentation) |
| 27 | 55 | c.218_219ins22,p.Ile223Thr c.218_219ins22,p.Ile223Thr | NA | NA | ？ | M | 7.2 | 十 | 十(facial dysmorphism, woolly hair) |  | 十 |  |  | 十 |  |  |
| 27 | 56 | c.668T>C,p.Ile223Thr c.829G>T,p.Glu277 | NA | NA | NA | F | 2.4 |  | 十(pectus excavatum fish mouth, fine and brittle hair) | 十 | 十 |  |  |  |  |  |
| 27 | 57 | c.977T>C,p.Ile326Thr c.977T>C,p.Ile326Thr | NA | NA | NA | F | 19.2 |  | 十(pectus excavatum fish mouth, fine and brittle hair) | 十 | 十 |  |  |  |  |  |
| 35 | 58 | NA | China | No | No | M | 480 | 一 | 一 | 一 | 一 | 一 | 一 | 一 | 一 | 一 |
| 33 | 59 | c.295C>T,p.Arg99Trp c.295C>T,p.Arg99Trp | Spanish | No | No | M | 14 | 十 | 十(thin skin with an absence of body hair and very thin eyebrows,limited mouth opening,high and arched palate,numerous dental caries,small and dystrophic nails) | 一 | 一 | 十(canker sores  facial erythema,skin lesions affecting the trunk and upper extremities) | 十 | occasional diarrhoea |  | 十(cataracts) |
| 10 | 60 | c.948-949delAAinsGG,p.Lys317Glu c.948-949delAAinsGG,p.Lys317Glu | Syrian | No | No | M | 5 | 十 | 十(brittle ashcolored hair) | 十 |  | 十(oral mucositis, swelling and erythema in his wrists and ankles) | 一 | abdominal discomfort | 一 | 一 |
| 30 | 61 | NA |  |  |  | M |  |  |  |  |  |  |  |  |  |  |
| 30 | 62 | NA |  |  |  | M |  |  |  |  |  |  |  |  |  |  |
| 32 | 63 | homozygous mutation |  |  |  | F | 36 | 一 |  |  |  | 一 | 十 | 十 |  |  |
| 28 | 64 | c.495_498del,p.F167Tfs*9 c.1246A>G,p.K416E | Latin American | No | No | F | 3 | 十 |  | 十 | 十 | 十(panniculitis) | 一 |  |  |  |
| 29 | 65 | c.1057-7C>G,? c.1092A>T,? |  |  | Yes(his sister had similar but milder features) | M | 5 | 十 | 十(disproportionally large head,coarse and thinning hair) | 十 | 十 |  | 十 | 一 |  |  |
| 9 | 66 | c.448C>T,p.R150C ? |  |  | No | F | 3 | 十 |  |  |  | 十(intermittent erythema accompanied with swelling of both hands, feet, knees and face) |  |  |  |  |
| 31 | 67 | c.977T>C,p.Ile326Thr  c.977T>C,p.Ile326Thr | African | Yes | Maybe(had a older sister who deceased at 29 weeks of gestation with intrauterine growth restriction, severe anaemia and respiratory and cardiac failure without DNA testing) | F | Soon after birth | 十 |  |  | 十 |  |  | 十 |  |  |
| 34 | 68 | heterozygous TRNT |  |  |  |  |  | 十 |  |  | 十 | 十 |  | GI bleeding |  |  |
| 35 | 69 | c.668T>C,p.Ile223Thr  c.668T>C,p.Ile223Thr | Portuguese |  |  | M | 8 |  |  |  | 十 |  |  | malabsorption and TPN dependence |  | 十(optic atrophy，pigmentary retinopathy) |
| this article | 70 | c.706G>A,p.Glu236Lys c.706G>A,p.Glu236Lys | China | No | Maybe(had a similarly affected older sister who died at 2 years old without WES) | M | 1.5 | 十 |  |  | 十 | 十(recurrent eczema) |  | 十 |  |  |
| this article | 71 | c.907C＞G,p.Gln303Glu c.88A＞G,p.Met30Val | China | No | No | F | 17hours | 十 | 十(mild facial dysmorphism) | 十 | 十 | 十(erythema on the face, trunk and limbs, skin pathology showed part of the epidermis was denatured and necrotic) | 十 | 十 |  |  |

**Continued**

| References | Patient | Musculoskeletal involvement | Metabolic and endocrine abnormality | Cardiomyopathy | Neurological manifestations | Infection | Elevated inflammatory markers | Hypogammaglobulinaemia | B lymphopenia | Microcytic anemia/ Sideroblasts anemia | Brain MRI | others | Treatment and effect | outcome |
| --- | --- | --- | --- | --- | --- | --- | --- | --- | --- | --- | --- | --- | --- | --- |
| 6 | 1 | 十(hypostenic muscle) | 十(metabolic acidosis, dyselectrolytemia,isolated growth hormone deficiency, anterior pituitary hypoplasia) | 十 |  |  | 十 | 十 |  | 十/- |  |  | 1.corticosteroid: moderately controlled  2.ACE inhibitors, β-blockers, digitalis, diuretics and acetylsalicylic acid: improvement at echocardiography  3.anakinra: no effect 4.etanercept: good response 5.GH: partial improvement |  |
| 6 | 2 | 十(arthritis of the right ankle) |  |  |  | 一 | 十 |  |  |  | 一 |  | 1.anakinra: partial response 2.etanercept: good response 3.GH |  |
| 14 | 3 |  | 十(intermittent hypoglycemia) |  |  | 十(recurrent methicillin-resistant Staphylococcus aureus (MRSA) skin infections) |  | 十 | 一 | 十/-(α thalassemia） |  |  | GH: resolve hypoglycemia, but suboptimal impact on her height |  |
| 14 | 4 |  |  |  |  |  |  |  | 一 | 十/-（α thalassemia） | prominent ventricles and subarachnoid spaces |  | 1.GH: suboptimal impact on his height 2.cochlear |  |
| 15 | 5 | 十(joint hyperlaxity, elevation of creatin kinase 2083IU/l) |  |  |  |  |  | 十 | 十 |  | nonspecific white matter hyperintensity on T2 brain MRI |  | IVIG replacement: have responds |  |
| 16 | 6 | 十(non-pitting edema of the backside of both his hands, ankle joints, and the dorsum of his foot, symmetrical swelling of bilateral wrists and knee and ankle joints, lexion contracture in the knee joint) |  |  | 十(high muscle tension in the left lower limb) | 十(respiratory tract infection) |  | 十 |  | 十/- |  |  |  |  |
| 16 | 7 |  |  |  |  |  |  | 十 |  | 十/- |  |  |  |  |
| 17 | 8 |  |  |  |  | 十(CMV) | 十 | 十 | 十 | 十/- |  | increase surface Interleukin 2 receptor, Interleukin 6, Interleukin 8, TNFalpha during febrile episodes | 1.short-term prednisone  2.IVIG replacement | The frequency of febrile episodes decreased over time, and the time between attacks increased to 3-4 months |
| 3,5,12 | 9 | 十(muscle biopsy demonstrated markedly reduced complex IV activity expressed as a ratio to citrate synthase at 0.007 (0.014–0.034)) | 十(aminoaciduria±hyperalaninemia，lactic acidosis, dyselectrolytemia, generalised aminoaciduria, elevated proline 347 μmol/L (85–290), alanine 749 μmol/ L(150–450) and glutamine 1014 μmol/L (480–800) of plasma, strongly raised 3-hydroxybutyrate and acetoacetate of urine, mildly raised urate) |  |  |  |  | 十 | 十 | 十/十 | cerebral atrophy | urate renal calculus which resulted in right sided hydronephrosis, abnormal high amplitude slow activity on EEG | 1.regular transfusion 2.IVIG replacement | She dead at 14years old because of Sepsis with multiorgan failure and toxic epidermal necrolysis (attributed to a cephalosporin) |
| 3,5,12 | 10 |  | 十(aminoaciduria±hyperalaninemia， lactic acidosis，increased urinary TCA intermediates, mildly raised pyruvate of urine) |  | 十(hypotonia) |  |  | 十 | 十 | 十/十 | cerebral atrophy | nephrocacinosis | 1.IVIG replacement  2.BMT: his fevers resolved, his growth improved and he made some developmental progress. He remains systemically well 3 years post BMT. At 10.5 years of age he has started to walk independently and is making progress with speech, but continues to have moderate hearing loss and retinopathy | His fevers resolved, his growth improved and he made some developmental progress after BMT at 7 years old. He remains systemically well 3 years post BMT. At 10.5 years of age he has started to walk independently and is making progress with speech, but continues to have moderate hearing loss and retinopathy |
| 3,5 | 11 |  | 十(aminoaciduria±hyperalaninemia，renal tubular Fanconi syndrome) |  | 十(seizures) |  |  | 十 | 十 | 十/十 | cerebral atrophy, decreased cerebellar perfusion |  | 1.regular transfusion 2.IVIG replacement | She dead at 61 months because of multiorgan failure |
| 3,5 | 12 |  |  |  | 十(seizures) |  |  |  |  | 十/十 | communicating hydrocephalus, macrocephaly | pancreatic insufficiency | regular transfusion | He dead within hours because of arrived in shock with severe hypoglycemia and multiorgan failure at 56 months |
| 3,5 | 13 |  |  | 十 | 十(ataxia/cerebellar signs) |  |  | 十 | 十 | 十/十 |  |  | 1.regular transfusion 2.IVIG replacement | She dead at 25 months because of cardiac failure secondary to cardiomyopathy (not related to iron overload) |
| 3,5 | 14 |  | 十(aminoaciduria±hyperalaninemia) |  | 十(ataxia/cerebellar signs, seizures) |  |  | 十 | 十 | 十/十 |  |  | 1.regular transfusion 2.IVIG replacement | He dead at 16 months because of multiorgan failure (pneumonitis, cardiac failure) |
| 3,5 | 15 |  | 十(aminoaciduria±hyperalaninemia, hypercaciuria) |  | 十(ataxia/cerebellar signs, seizures) |  |  | 十 | 十 | 十/十 | cerebral atrophy, abnormal enhancement of external capsule and thalamus |  | 1.regular transfusion 2.IVIG replacement |  |
| 3,5 | 16 |  | 十(aminoaciduria±hyperalaninemia) |  | 十(ataxia/cerebellar signs, delayed white matter myelination) |  |  | 十 | 十 | 十/十 | delayed white matter myelination | nephrocacinosis |  |  |
| 3,5 | 17 |  |  | 十 |  |  |  | 十 | 十 | 十/十 |  | hepertension | 1.regular transfusion 2.IVIG replacement | She dead at 28 months because of cardiac failure secondary to cardiomyopathy (not related to iron overload) |
| 3,5 | 18 | 十(nonspecific metabolic myopathy on muscle biopsy) | 十(aminoaciduria±hyperalaninemia, lactic acidosis) |  |  |  |  | 十 | 十 | 十/十 |  |  | 1.regular transfusion 2.IVIG replacement 3.BMT at 9 months old: alive(4 years) |  |
| 3,5 | 19 |  |  |  |  |  |  |  |  | 十/十 |  |  | regular transfusion |  |
| 5 | 20 |  |  |  |  |  |  |  |  |  |  |  | 1.regular transfusion 2.IVIG replacement 3.BMT at 9 months old | The patient dead at 10 months old because of pulmonary hemorrhage post BMT |
| 5 | 21 |  |  |  |  | 十 |  |  |  | 十/NA |  |  | IVIG replacement |  |
| 5 | 22 |  |  |  |  | 十 |  |  |  | 十/NA |  |  | IVIG replacement |  |
| 5 | 23 |  |  |  |  |  |  |  |  |  |  |  | 1.regular transfusion 2.IVIG replacement |  |
| 5 | 24 |  |  |  |  |  |  |  |  |  |  |  | 1.regular transfusion 2.IVIG replacement |  |
| 8 | 25 | 十(knee arthritis) |  |  | 十（vertigo, febrile seizures） | 十 | 十 | 一 | 一 | 十 |  |  | 1.systemic corticosteroids: partial response  2.colchicine: partial response  3.blood transfusions 4.empiric antibiotics | She died of multiorgan failure at the age of 7 years during a sepsis-like culture-negative febrile episode |
| 8 | 26 |  |  |  | 十(dizziness/vertigo, opsoclonus) | 十(upper respiratory symptoms, wheezing, Avian Influenza A virus infection | 十 | 一 | 一 | 十/十 |  |  | 1.GH replacement therapy 2.blood transfusions 3.systemic corticosteroids 4.empiric antibiotics 5.colchicicine 6.etanercept |  |
| 8 | 27 |  |  |  | 十(hypotonia, seizures, suspected CNS MAS) | 十(Acinetobacter ursingii sepsis, Escherichia coli acute pyelonephritis, Staphyloccocus aureus sepsis) | 十 | 十 | 十 | 十/十 | end stage cerebral damage, hemorrhage, volume loss and leukomalacia | elevated liver enzymes,  preterm delivery and perinatal complications; history of thrombosis of femoral central vein catheter | 1.blood transfusions 2.systemic corticosteroids 3.empiric antibiotics 4.cyclosporin A 5.IVIG replacement 6.BMT | He had prominent neurological deficits and severe B cell immunodeficiency, died at the age of 3 years, 92 days post BMT |
| 8 | 28 | 十(elevated muscle enzymes) |  |  | 十(intermittent opsoclonus and nystagmus) | 十(cellulitis of both feet and face, Nasal congestion, rhinorrhea, sinusitis, Enteroviral gastrointestinal infection, Clostridium difficile colitis, bacterial urinary tract infections, Respiratory syncytial virus pneumonia) | 十 |  |  | 十/十 | 一 | transient pancreatic insufficiency,  elevated liver enzymes. hemophagocytes on bone marrow aspirate smear | 1.blood transfusions 2.empiric antibiotics 3.IVIG replacement  4.anakinra  5.etanercept |  |
| 8 | 29 | 十(arthritis of medium and large joints, proximal muscle weakness, muscle biopsy showed acute myositis/fasciitis and inflammatory infiltrates by lymphocytes and macrophages ) |  |  | 十(mild ataxia and balance difficulties, absent reflexes at the ankles) | 十(cellulitis after minor trauma, recurrent croup, stridor and epiglottitis) | 十 | 十 | 十 | 十/一 |  | bilateral small kidneys on ultrasound | 1.empiric antibiotics 2.steroids 3.IVIG replacement  4.etanercept switched to infliximab and azathioprine due to development of inflammatory colitis 5.cochlear implants |  |
| 8 | 30 |  |  |  |  | 十(recurrent otitis media, urinary tract infection, sinusitis, pneumonia) |  | 十 |  | 十/十 |  | attention deficit disorder, asthma | 1.blood transfusions  2.IVIG replacement |  |
| 8 | 31 |  |  |  |  | 十(recurrent otitis media, pneumonia，Staphyloccocus aureus septic shock) |  | 十 |  | 十/十 | pseudotumor cerebri | central catheter-related venous thrombosis of superior vena cava, nodular regenerative hyperplasia and hemosiderosis seen in liver autopsy postmortem, asthma | 1.anticoagulation for DVT 2.splenectomy for transfusion dependent anemia  3.blood transfusions 4.IVIG replacement  5.steroids  6.NSAIDs | She died at the age of 9 years from Staphylococcus aureus sepsis |
| 8 | 32 |  |  |  |  | 十(recurrent rhinitis and cough, Rotavirus gastroenteritis) | 十 | 十 | 十 | 十/十 |  |  | 1.IVIG replacement  2.anakinra for 3 months: lack of effect on fever episodes 3.cochlear implants |  |
| 8 | 33 |  | 十(hypoglycemia) |  |  | 十(CMV viremia, CMV pneumonitis, Pseudomonas necrotizing perianal colitis) |  |  | 十 | 十/十 |  | pancreatic insufficiency | 1.blood transfusions  2.IVIG replacement  3.steroids 4.etanercept  5.hearing aids |  |
| 13 | 34 |  |  | 十 | 十(intraventricular haemorrhage, white matter necrosis, haemorrhagic foci within the basal ganglia) |  |  |  |  | 十/- |  | poor response to resuscitation requiring endotracheal intubation, jaundice, abdominal distension, peritoneal, pericardial and pleural effusions, haemorrhagic petechiae on the anterior and posterior aspects of the heart and subpleural areas, adrenals, liver and spleen capsule, the placenta was hydrops, large, pale and friable, histology revealed marked villous hydrops and generalised villous immaturity, and diffuse extramedullary haemopoiesis with large clumps of erythropoietic cells and myeloid precursors within, plugging the fetal vessels, the chorionic plate vessels and the umbilical cord vessels | blood and fluid resuscitation | She dead with progressive jaundice, multiorgan failure and intracranial bleeding，the patient died at around 40 hours of age |
| 13 | 35 |  |  | 十 |  |  |  | 十 |  | 十/十 |  | short long bones, a possible cardiomyopathy and dilated bowel loops were seen on ultrasound from 20 week in pregnancy, persistent but stable fetal tachycardia was noted at 30 weeks, poor cardiotocograph traces after birth mild proximal upper limb shortening and penoscrotal hypospadias and microphallus, conjugated hyperbilirubinaemia, adrenal calcification | 1.transfusion support-then became transfusion dependent 2.BMT at the age of 5 months | The patient developed significant neurological complications soon after transplantation, including intractable seizures, with white matter and subsequently cystic changes on MR. Following admission to paediatric intensive care, he experienced a significant deterioration in his clinical status, including idiopathic pulmonary syndrome and ventilator dependence. The patient died peacefully on day 38 following transplant |
| 11 | 36 | 十(joint swelling of fingers, elbow and knees) |  |  | 十(low muscle tone, poor balance) |  | 十 | 十 | 十 | 十/一 |  |  | 1.blood transfusions 2.IVIG replacement 3.anakinra: episodes were not completely controlled 4.anakinra**+**colchicine: significant improvement in his symptoms 5.etanercept：fevers recurred 6.etanercept+colchicine: improved control of fever episodes and improvement of inflammatory index and haemoglobin level |  |
| 4 | 37 | 十(arthritis） |  |  |  | 十(herpetic stomatitis, tonsillitis, lobar pneumonia, Metapneumovirus tracheitis) | 十 | 十 |  | 十/NA |  | lymphadenopathy, premature newborn, ductus arteriosus | 1.blood transfusions  2.subcutaneous immunoglobulin 3.etanercept: improve 4.cochlear implantation | The fever, anaemia, skin neutrophilic infiltration and the inflammatory parameters improved |
| 7 | 38 | 十(arthritis） |  |  |  |  | 十 | 十 | 十 | 十/- |  |  | 1.IVIG replacement  2.sterodis 3.etanercept: control of fever, skin and osteoarticular symptoms |  |
| 18 | 39 | 十(osteoporosis) | 十(hypothyroidism) |  |  |  |  | 十 |  | 十/十 |  |  | 1.IVIG replacement  2.topical steroids |  |
| 19 | 40 | 十(hip and neck arthritis) |  |  |  |  |  |  |  | 一/一 |  | abnormally small red blood cells and significant anisocytosis，The peripheral blood smear revealed microcytosis, hypochromia, anisocytosis and poikiocytosis with numerous eliptocytes | methotrexate |  |
| 19 | 41 | 一 |  |  |  |  |  |  |  | 一/一 |  | a low normal hematocrit, microcytosis and anisocytosis |  |  |
| 19 | 42 | 一 |  |  |  |  |  |  |  | 一/一 |  | microcytosis and an elevated erythrocyte distribution width |  |  |
| 20 | 43 |  | 十low IGF-1 and IGF-1BP3 with a mild increase on IGF-1 generation test, estrogen and AMH undetectable, LH pubertal level and FSH raised at baseline and post LHRH stimulation, prepubertal uterus with no ovaries visualized in pelvic ultrasound, primary ovarian failure |  | 十(poor balance) | 十(hepatitis A) |  | 十 | 一 | 十/一 |  | bronchiectasis | 1.IVIG replacement  2.GH 3.low-dose estrogen therapy  4.sequential cataract surgery with intraocular lens implantation |  |
| 20 | 44 |  |  |  | 十(poor balance) |  |  | 十 | 一 | 十/一 |  |  | 1.IVIG replacement  2.surgery with intraocular lens implantation |  |
| 20 | 45 |  |  |  |  |  |  | 十 | 一 | 十/一 |  | bronchiectasis | 1.IVIG replacement  2.right cochlear implant surgery  3.both eyes underwent cataract surgery with intraocular lens implantation |  |
| 21 | 46 |  |  |  |  | 十(upper respiratory tract infections, bronchitis) |  | 十 |  | 十/十 |  | nonspecificpulmonary nodules on CT | 1.IVIG replacement: frequency of respiratory tract infections decreased, Ig levels gradually increased |  |
| 22 | 47 |  | 十(low levels of sex and growth hormones) |  |  | 十(pneumonia) |  | 十 | 十 | 一/一 |  |  | IVIG replacement |  |
| 23 | 48 |  |  |  |  | recurrent septicaemias | 十 | 十 | 十 | 十/一 |  | vulvitis, parotitis, adenitis,progressive lymphopenia with undetectable levels of B lymphocytes and CD27+ B memory cells by age 15 months,increased IFN-α protein,increased expression of ISGs,a negative IFN score on one occasion | IVIG replacement | At 6 years of age, her height was and development were normal, and no infections had occurred,She continued to have 3to 4 febrile attacks (lasting for 1 to 2 days) each month associated with elevated CRP levels anemia resolved spontaneously |
| 24 | 49 | 十(muscle biopsies showed increased numbers of enlarged mitochondria on electron microscopy) | 十(lactic acidosis，chronic mild metabolic acidosis, hyperlactatemia, hyperalaninemia, renal Fanconi syndrome) |  | 十(seizures, central apnea at 18 months of age) |  |  |  |  | 十/十 | acquired microcephaly with cortical atrophy | increased numbers of enlarged mitochondria on electron microscopy of liver and kidney biopsies |  | She died of cardiac arrest during an episode of fever and acute acidosis at 21 months of age |
| 24 | 50 |  |  |  | 十(gait ataxia, dysarthria, gross motor regression, hypotonia, ptosis and horizontal ophthalmoplegia, slow speech) |  |  |  |  |  | abnormal signals in brainstem and dentate nucleus |  |  |  |
| 25 | 51 |  |  |  |  | 十(bronchitis, pneumonia,invasive enterocolitis caused by Campylobacter jejuni) |  | 十 | 十 |  |  | low isohemagglutinin titer | 1.IVIG replacement 2.surgery for bilateral cataracts 3.corticosteroid: recurrence of bloody diarrhea was successfully treated,but corticosteroid was discontinued only after 1 year because of several relapses of diarrhea 4.azathioprine and otilonium bromide: his gastrointestinal symptoms are controlled |  |
| 26 | 52 |  |  | 十 |  | 十(Klebsiella oxytoca urinary tract infection) |  | 十 |  | 十/十 |  |  | 1.IVIG replacement 2.steroid | He suffered from sepsis-like episode complicated by multi-organ failure and died shortly thereafter |
| 12 | 53 |  | 十(metabolic acidosis, electrolyte imbalance) |  | 十(recurrent episodes of encephalopathy associated with nystagmus and photophobia) | 十(recurrent bacterial infections) | 十 | 十 | 十 | 十/十 | subsequently progressive cerebellar atrophy, widespread abnormalities with multiple lesions resembling infection in the last stages of her disease | thrombocytopaenia, pancreatic insufficiency | 1.IVIG replacement 2.benzodiazepine | She died at 3 years and 3 months following a progressive encephalopathy |
| 12 | 54 |  |  |  | 十(central hypotonia, seizures) |  |  | 十 | 十 | 十/一 | multiple focal lesions in the cerebral hemispheres and cerebellum | pancreatic insufficiency |  | He died at 10 months of age because of intractable status epilepticus |
| 27 | 55 |  | 十(metabolic acidosis) |  | 十(nystagmus) |  | 十 | 十 |  | 十 |  |  | transfusion support | Died |
| 27 | 56 |  |  |  | 十(hypotonia) |  |  | 十 |  | 十/一 |  |  | transfusion support | Died |
| 27 | 57 |  |  |  |  |  |  | 十 |  | 十/一 |  |  | transfusion support |  |
| 35 | 58 | 一 | 一 | 一 | 一 | 十(sever and recurrent COVID-19（SARS-CoV-2） | 一 | 十 |  |  | 一 | Insufficient production of protective antibodies | 一 |  |
| 33 | 59 | 十(joint redness,hand swelling) | 十(primary hypogonadism,  low testosterone levels, elevated gonadotropins and luteinizing hormone →hypergonadotropic hypogonadism,reduced size of the penis and testicles, autoimmune thyroiditis) | systolic murmur | 十(peripheral facial palsy) | 十(chickenpox,mumps,urinary tract infection,bronchitis,pneumonia,otitis) |  | 十 | 十 | 十/NA |  | Decreased CD3+T,CD3+CD4+T,CD3+CD8+T and B lymphocytes | IVIG | Unspecific fever，canker sores, and rash disappeared at 6 years of age |
| 10 | 60 | 十(swelling and pain on the dorsum of his hand,arthritis of the first metatarsophalangeal joint) | 十(GH deficiency) | 一 | 一 | 十(recurrent pneumonia and diarrhea) | 十 | 十 | 一 | 十/NA | 一 | 一 | 1.low-dose corticosteroid:partial response 2.Colchicine:no response to colchicine treatment 3.monthly intravenous immunoglobulin replacement 4.weekly subcutaneous etanercept:good response | Monthly intravenous immunoglobulin replacement and weekly subcutaneous etanercept resolved fever episodes,infections occurred,and improved anemia and growth parameters |
| 30 | 61 |  |  |  |  |  |  |  |  | 十/十 |  |  |  |  |
| 30 | 62 |  |  |  |  |  |  |  |  | 十/十 |  |  |  |  |
| 32 | 63 | 一 | 一 | cardiac arrest | 十(complex febrile seizures, acute disseminated encephalomyelitis,epileptic encephalopathy,brain infarctions) | 十(recurrent viral gastroenteritis,pneumonias-rhinovirus) |  | 十 |  | hereditary pyropoikilocytos | subcortical infarcts and periventricular ischemia | severe hemolytic anemia | 1.steroids:control seizures,improved epileptic encephalopathy,cardiac arrest,and brain infarctions 2.SCIG:infections improved | SCIG was discontinued at age 9 with subsequent evaluation revealing normal immunoglobulin levels Hearing loss persisted Final admission was complicated by status epilepticus and severe hypoxemia leading to brain death Autopsy showed cerebral edema with tonsillar herniation, encephalomalacia, hemorrhages, and non-perfused brain |
| 28 | 64 |  |  |  |  | 十(herpes stomatitis, tonsilitis, lobar pneumonia, viral tracheitis due to Metapneumovirus) | 十 | 十 |  | 十/NA |  | premature baby, 34 weeks, 2.140 g, with laryngomalacia.respiratory distress after birth,fever and local reaction after each vaccination. Local reaction was reported after each | blood transfusion |  |
| 29 | 65 |  |  |  |  |  |  |  |  | 十/十 |  | elevated liver enzymes,jaundice,growth hormone deficiency,peripheral blood smear showed marked anisopoikilocytosis including target cells, elliptocytes, tear drops, spherocytes, poikilocytes, marked polychromasia, and coarse basophilic stippling. |  |  |
| 9 | 66 | 十(swelling of both hands, feet, knees) |  |  | 十(right-sided hemiconvulsions,dysrhythmic EEG changes,seizures) |  | 十 | 一 | 一 | 十/一 |  | progressive fatigue | 1.short-term methylprednisolone: the frequent and prolonged febrile episodes were successfully treated but failed to prevent the occurrence of new episodes 2.TNFα inhibitor(etanercept): no further febrile episodes or seizures and improvement in appetite and general condition | The child had no further febrile episodes or seizures and there was an improvement in appetite and general condition after the initiation of etanercep |
| 31 | 67 |  |  |  |  |  | 十 |  | 十 | 十/十 |  | IUGR,T CD8+ and NK lymphopenia,B cell defect,increased IFN-α protein,increased expression of ISGs,STAT1 and STAT3 were constitutively phosphorylated in T CD3+ lymphocytes and monocytes,a negative IFN score on one occasion | blood transfusion | Sideroblastic anaemia resolved spontaneously at the age of 4 years |
| 34 | 68 |  |  |  |  | 十(frequent bronchitis) |  |  |  |  |  |  | 1.anakinra:failed 2.IVIG:successful 3.colchicine:partial response |  |
| 36 | 69 |  | hypernatremia |  | Leigh encephalopathy, spasticity, dystonia, and focal dyscognitive seizures | recurrent infections (pulmonary, gastrointestinal) |  |  |  | 十/十 | symmetric hemorrhagic lesions in the thalamus, brain stem (periaqueductal grey) and cerebellum consistent with Leigh syndrome but no lactate peak on MRS. | pancytopenia  tracheostomy dependence |  | Survival to early adulthood |
| this article | 70 |  | 十(metabolic acidosis) |  |  | 十(Adenovirus(Sputum), Klebsiella pneumoniae (sputum and urine), Clostridium difficile colitis, pneumonia) | 十 | 十 | 十 | 十/一 | widen of bilateral ventricles, occipital cistern and left cerebellar extracerebral space | normal T lymphocytes, decreased B lymphocytes, increased NK cells,elevated IL-6 | 1.antibiotic 2.IVIG replacement |  |
| this article | 71 |  |  |  |  | 十(pneumonia-Klebsiella pneumoniae) | 十 | 十 | 十 | 十/一 | abnormal white matter signals in bilateral frontotemporal parietal occipital lobes, banded abnormal signals near bilateral lateral ventricles | increased T lymphocytes and NK cells, B lymphopenia, elevated IL-6, IL-8, and IFN-γ levels. significant increase in γδT cells  elevated ALT,thrombocytopenia | 1.transfusion support 2.antibiotic 3.IVIG replacement 4.steroiled: partincal improved 5.adalimumab: partincal effective 6.thalidomide: effectively control fever, vomiting and diarrhea, improve anemia |  |

*“＋” indicated that the patient had vomiting and diarrhea in the column of “Gastrointestinal involvement”; **the patient had a similarly affected sibling without WES. Abbreviations: ACE: angiotensin converting enzyme; WES: whole-exome sequencing; OU: oculus unati; GH: growth hormone; GI: gastrointestinal; BMT: boon marrow transplantation; IVIG: intravenous immunoglobulin; DVT: deep venous thrombosis; NSAIDs: nonsteroidal anti-inflammatory agents; MRSA: methicillin-resistant *Staphylococcus aureus*; SCIG: subcutaneous immunoglobulin; IUGR: intrauterine growth retardation; NK: natural killer; ISGs: IFN stimulated genes; NA: not available
